# Intellectual property protection for traditional medical knowledge in China’s context: a round peg in a square hole?

**DOI:** 10.1093/medlaw/fwad006

**Published:** 2023-04-05

**Authors:** Nan Xia

**Affiliations:** Faculty of Business and Law, Queensland University of Technology, Brisbane, Australia

**Keywords:** China, Intellectual property, Legal transplanting, Local communities, Patent law, Traditional medical knowledge

## Abstract

This article is an examination of the extent to which traditional medical knowledge in China can be protected by intellectual property laws. The analysis begins by providing a global picture with regard to the historic origin of intellectual property, exploring the reasons why China does not have indigenous counterparts to the western system of intellectual property rights protecting its traditional knowledge (including traditional medical knowledge) and stating the problems of transplanting western intellectual property standards in China. A discussion follows on how China, under foreign pressure, has made efforts to comply with the changing standards mandated by various international, regional, and bilateral arrangements related to intellectual property, with examples of the development of China’s patent law. China’s approach towards the protection of traditional medical knowledge in various international fora related to intellectual property is explored. Finally, there is a specific examination of the compatibilities between the western system of intellectual property rights and traditional medical knowledge in China, at the national and community levels. This article argues that the system of intellectual property rights does not easily fit with China’s traditional medical knowledge because of China’s unique cultural traits, distinctive historical context and wide ethnic, religious, and local community diversity.

## I. INTRODUCTION AND BACKGROUND

‘How is the Owner of “TChina is a land of enduring traditions, which has gathered knowledge over millennia.^1^ Chinese traditional knowledge presents particular social and cultural values and plays a vital role in the daily lives of China’s national, sub-national, and local communities. For instance, as powerful symbols of China’s nationalist identity and the cultural values handed down as ancestral wisdom, traditional medical knowledge (TMK) has maintained its popularity in all regions of China. Indeed, it has been suggested that TMK accounts for between 30% and 50% of Chinese health care.^2^

Nevertheless, despite the significance of TMK in China, such knowledge within various dominant legal and social discourses has long been considered to be raw, archaic and lacking in modern commercial values.^3^ This negative classification of TMK marginalises the communities and holders of TMK and could open them up to adverse effects, including the misappropriation of TMK for exploitation and commercialisation, and its values being lost in the complementary processes of globalisation and modernisation. On this basis, there have been calls for China’s TMK to be protected, not merely as intangible property but also, and more significantly, as a matter of historical and cultural significance to the dignity and cohesion of Chinese national, sub-national, and local communities.^4^

Such calls appear to resonate within intellectual property (IP) systems and are informed by the commercial and trade value of TMK. However, due to the differences between perceptions about TMK from a traditional Chinese perspective and a ‘western’ legal perspective, an IP framework could be an unsuitable legal framework for the protection of TMK in China’s unique cultural and historical context. The ‘round peg in a square hole’ analogy illustrates this conflicting situation.^5^ The following questions therefore arise: to what extent are the existing IP regime and TMK system compatible with each other in China’s context? How should the IP system be improved—legally and in practice—to ensure that the IP system functions better to accommodate the characteristics of the TMK system in China’s context? In this article, these issues are examined and recommendations are formulated for reforming the legal frameworks in China. Through the analysis, it is argued that the patent-based system of IP rights sits uneasily with TMK in China. This is due to China’s unique cultural traits, distinctive national traditions, and wide ethnic, religious, and local community diversity.

This article is structured into three sections. Following this introduction and background, in Section II the historical origin of IP laws in the international context and the historical process of adaptation and rejection of transplanted IP systems in China is outlined, and their wider implications and associations with the TMK system in China are discussed. Section III consists of an examination of the Chinese approach to complying with the changing standards mandated by various international, regional, and bilateral arrangements related to IP, and of China’s specific approach towards the protection of TMK in various international fora related to IP. In the final section, the compatibility and level of protection offered by the Chinese IP system to TMK in China’s domestic context is assessed, and recommendations for reforms are provided.

Internationally, the term ‘traditional knowledge’ (TK) has been adopted under various settings, particularly in debates on biodiversity, human rights, and health.^6^ Although there is no agreed definition and formal classification of TK, various efforts have been made to categorise it. Generally speaking, TK can be divided into three categories: TMK, traditional agricultural knowledge, and traditional ecological knowledge.^7^ In this article, and to provide an in-depth analysis, I will mainly focus on TMK rather than discuss TK as a whole. This is important because TMK has a significant role in maintaining and enhancing health in China.

Despite the tripartite classification of TK, TMK itself is difficult to define. This is largely because its definition varies according to different cultures, languages, and geographic locations across the world. In China, TMK is legally defined as:

The total body of medical knowledge of the various ethnic groups in China, including the Han ethnic group and other ethnic minority groups, that reflects China’s various ethnic groups’ understanding of life, health and illness, and are based on China’s long historic tradition, unique theory and technical method.^8^

This definition is specific and focuses on the type of medical knowledge used by knowledge holder communities in China, including the ‘Han ethnic group’ and ‘other ethnic minority groups’ as independent knowledge systems to treat a variety of diseases. Such a definition carries the spirit of TMK by recognising its essential characteristics: its long historical tradition of being passed from one generation to the next, and a clarification of who the right holders of TMK in China are. This definition was created in the context of China’s unique social, cultural and historical elements.

In contrast, the World Health Organization’s (WHO) definition of TMK is broader and includes:

diverse health practices, approaches, knowledge and beliefs incorporating plant, animal and/or mineral-based medicines, spiritual therapies, manual techniques and exercises applied singularly or in combination to maintain well-being, as well as to treat, diagnose or prevent illness.^9^

This definition reflects the holistic nature of TMK as it harmonises various traditional medical practices with cultural values, beliefs, and spiritual dimensions concerning the significance of illness, healing, and health. Interestingly, China’s TMK system also contains holistic features as it is assumed that nature, society, and humans are bonded together by something undetectable.^10^ Although the WHO’s definition does not include elements unique to China, the openness of their definition, which provides for similar holistic medical patterns and diverse methods of preparation of traditional medicine, makes it applicable to the analysis in this article. Therefore, the Chinese government’s and the WHO’s definitions together can provide a framework for the discussions to follow.

In this article, I acknowledge that TMK is dynamic and evolving rather than static and fixed. Therefore, the protection of TMK should be envisaged as an incentive for further innovation and development of the traditional culture, and not just to protect ‘old’ information from being disclosed in the public domain. For centuries, the use of and transmission of TMK in China has been regulated by customary rules, practices, and traditions.^11^ These have evolved throughout the generations and adapted to the changing circumstances and requirements of local communities and the state as a whole. I also recognise that TMK has been nurtured and evolved over centuries by local communities in China, and that TMK stems from complicated and dynamic knowledge systems intricately linked to cultures, livelihoods, and places it is intimately connected to.

IP laws, as one of the most significant frameworks used by the countries of the world to allocate rights over knowledge, play a critical role in protecting TMK and its associated genetic resources, and in ensuring that benefits from the use of TMK can be shared in a fair and equitable manner among the stakeholders.^12^ Since the 1980s, IP protection for traditional knowledge (including TMK) has been a subject of intense debates at various international fora.^13^ Many international organisations and institutions have since explored various international methodologies for protecting traditional knowledge within and outside the conventional IP system. For instance, the World Intellectual Property Organization (WIPO) has established an Intergovernmental Committee on Intellectual Property and Genetic Resources, Traditional Knowledge and Folklore (IGC), as a forum for WIPO Member States to discuss IP matters concerning the protection of traditional knowledge.^14^ The IGC has been active since 2000, based on a mandate to establish effective international legal instruments to protect traditional knowledge.^15^ The extensive efforts of the IGC have resulted in a range of information, practical strategies, and policy resources. This includes a gap analysis conducted in 2008 which is constantly updated to identify the shortcomings of the existing IP regimes and explore the options to address those shortcomings for traditional knowledge (including TMK) protection.^16^ These gap analyses have facilitated and supported the preparation of draft regulations for the protection of traditional cultural expressions/folklore and traditional knowledge against misappropriation.^17^

Since the early 2000s, there has been an explosion in the literature on IP protection and traditional knowledge from scholars within a range of disciplines.^18^ Nevertheless, substantial disagreements exist as to the general viability of linking traditional knowledge and IP protection, and how to fit traditional knowledge (including TMK) into certain accepted standards of western IP systems regarding originality, fixation, novelty, and duration.^19^ For example, supporters of IP protection believe that there is a case for adopting a properly constructed IP regime to protect traditional knowledge (including TMK).^20^ Indeed, Jim Chen has argued that the appropriate level of protection for TMK is provided by reliance on conventional IP law, and that only minor amendments would be needed in order to secure sufficient protection of TMK.^21^ Other scholars have argued that more significant reforms are required for the protection of TMK through IP-related system. For instance, Owen Dean has contended that a sui generis approach is required based on the existing IP system in order to accommodate the special characteristics of traditional knowledge (including TMK) and to satisfy the needs of traditional knowledge holders.^22^

Yet, others have viewed proposals for establishing IP protection for traditional knowledge with scepticism. Thus, Darrell Posey has argued that the IP system was developed in favour of individuals and not for the purpose of protecting the collective TMK of indigenous and local communities.^23^ Moreover, acquiring IP protection is prohibitively expensive for these communities. For instance, a patent from the China National Intellectual Property Administration costs a minimum of 27,000 Chinese yuan,^24^ so TMK holders from impoverished communities in need of food and other necessities are unlikely to fund such patent-based IP protection. Even though, in some cases, IP rights have been secured by TMK holders in communities, the cost for maintaining the IP assets and enforcing IP rights (when unauthorised exploitation of granted IP rights occurs) would price them out of any litigation and prosecutions using such instruments.^25^

These disagreements have prompted various attempts to harmonise IP protection. However, diverse interpretations and differences concerning IP protection for TMK still exist in different jurisdictions. This is due to the fact that each country has its own unique cultural traditions, social traits, historical and geographical specificities, identity and value systems.^26^ In the case of China, IP protection for TMK is special and complex, which deserves particular attention. This is because TMK in China represents the dynamic interactions among the scientific practices in western civilisation, the historically constructed knowledge system within eastern civilisation, as well as the knowledge or practices embedded within the traditions of local communities that form part of their cultural or spiritual identities. These distinctive features could make China’s TMK incompatible with a western IP regime. For instance, the Naxi communities of southwest China believe in a spiritual system, the Dongba culture, which aims to pursue the harmonious and balanced relationships between humans and their natural surroundings.^27^ Their practices of traditional medicine are derived from this culture and are characterised by deep spirituality and cultural beliefs.^28^ Specifically, as one famous TMK practice in the Naxi community, green thorn fruit oil is used not only as a cure for skin burns and scalds, but also as a means to avoid evil spirits. This reflects its extended meaning—worshipping the spirit of nature.^29^ This example demonstrates that it may not be possible for the cultural and spiritual features of the TMK system in China to be subject to IP protection. This is because while the technical and physical aspects of TMK can be protected by a western IP system, inherently spiritual and divine aspects of TMK may never be protected by IP. In this article, I argue that in the case of China, a system of IP rights, such as patents, sits uneasily with TMK because of China’s unique cultural traits and distinctive national traditions, as well as the variety and diversity of China’s TMK.

## II. THE EARLY GROWTH OF IP RIGHTS AND ITS INFLUENCE ON CHINA: A PROBLEMATIC LEGAL TRANSPLANT

### A. Historic origin and development of modern IP regime

It is widely acknowledged that the modern form of IP rights can be traced back to 15th century Europe when early modern science was emerging as an approach to investigating the natural world.^30^ The rise of capitalism and the development of new technologies, such as the printing press in the 15th century, contributed to the emergence of IP rights.^31^ Yet, the origin of the early concept of IP can be traced back further. Indeed, during the 12th and 13th centuries, medieval urban communities and free-market economies provided the impetus for the advent of a developed concept of IP, which was first present in the regulations of the guilds, where artisans and merchants were organised.^32^

Around that time, guilds were granted privileges to collect and transmit the particular knowledge inherited from the past and help protect the creations of others.^33^ Although the guilds never used the term ‘IP’, their assertion that they possessed the collective knowledge of their occupation as guild members, shows recognition of the intangible values derived from the rareness of knowledge.^34^ As their collective knowledge became increasingly valuable, individuals gradually broke away from the guilds system, taking their new views of guild knowledge with them and applying this knowledge to different interests.^35^ These clashes between the communal monopolies of the guilds and the novel individualised form of IP led to the emancipation of the individual knowledge holder and the concept of individual rights over intellectual inventions.^36^ Thus, it can be seen that the concept of IP as an individual right grew out of the medieval guild system, despite the term itself not being used until much later.

During the early centuries of IP development, the collective ownership of IP rights by members of collective guilds in Medieval Europe was similar to the collective or communal pattern of holding TMK in China. In the Chinese world view, familial and communal ties and collective benefits are held in high esteem.^37^ Likewise, TMK inherited from ancestors is usually considered as a collective benefit to the community and posterity. Nevertheless, and unlike in Europe where the concept of individual rights over intellectual creation arose, the ancient Chinese did not come up with a similar approach to recognise the creative individual’s IP rights.^38^ Nor did they consider the innovation of TMK to arise from the ingenuity of individuals.^39^ In general, TMK in China is considered to be commonly created and accumulated in a collective context, on the basis of the extensive exchange and circulation of ideas and information throughout generations. The collective ownership of such intangible TMK is deeply rooted in Chinese culture: there is the tacit agreement that no individual should have an exclusive entitlement to the collective TMK. This situation has thus created difficulties in breaking away from the established collective pattern in which TMK is held in China, and in attempting in China to create an indigenous counterpart to the western notion of IP rights.

Since the early 18th century, the western Industrial Revolution and rapid growth in the cotton, iron, and mining sectors increased the value of IP protection, providing impetus for the further development of the IP system.^40^ Accordingly, IP right holders started to look for protection of their home trade and industries, which led to a range of bilateral treaties, conventions, and agreements.^41^ Regardless of the content of these arrangements, the differences in the scope of domestic laws in different jurisdictions and differences in the treatment of nationals and foreigners caused serious challenges for those who wished to rely on such treaties, conventions, and agreements.^42^

This eventually resulted in the creation of the Paris Convention in 1883, which was the first international multilateral agreement on IP protection. Other significant international agreements for IP have followed, including treaties administered by the WIPO and the Agreement on Trade-Related Aspects of Intellectual Property Rights (TRIPs), administered by the World Trade Organization (WTO).^43^ These international agreements and treaties contain multilateral rules and apply to a range of parties.

Nevertheless, China did not embrace the development of IP protection in the way that its western counterparts did.^44^ This may have been because China was a self-sufficient feudally-based economy for thousands of years.^45^ This meant that the need for advanced science and technology did not exist, nor did developing the notion of IP into a solid framework.^46^ The 17th and 18th centuries witnessed the development of a notion towards IP in Europe whereby inventors could have a property interest in their inventions to defend against the state.^47^ In contrast, China continued to address the issues in this area mainly in terms of how best to maintain the state’s authority.^48^ Also, the country’s Confucian tradition, focusing on a collective orientation, was based on the responsibility of the seniors for the nurturing of their juniors. This made it even more impossible to consider the results of intellectual creations as private property.^49^

As a result, and unlike western countries, China had little of the industrial and technological progress that many scholars see as a catalyst for establishing IP systems. More significantly, Chinese traditional culture was Sinocentric and based on the concept of ‘the Celestial Empire’ and as China comprising the ‘centre of the world’.^50^ This Chinese centrality was based on the belief that all states in the world should arrange themselves hierarchically around the Chinese emperor who was known as the Son of Heaven.^51^ Thus, there was no notion of individual states’ legal equality or sovereignty.^52^ This means that such traditional cultural views do not fit into the notion of nations’ equal international relationship, the principle of territoriality and the national treatment principle laid down by international bilateral and multilateral agreements on IP protection.^53^ Therefore, this historical situation has created challenges for China in terms of embracing international IP-related agreements and implementing the relevant obligations.

### B. IP laws in Chinese civilization

There are no sustained indigenous counterparts to IP law in Chinese history before the introduction of a western standard of such law in the early 20th century.^54^ As noted by many scholars, Chinese legal history has long been characterised as ‘overwhelmingly penal in emphasis’, while barely concerning itself with civil matters pertaining to private property.^55^ For instance, prompted by the development of printing technology and a rise in literacy rates since the Song dynasty (960–1276), unauthorized reproduction and alteration of items in China increased over time. This phenomenon reached its peak in the 15th century, including the reproduction and alteration of items that were exclusively controlled by the state and those deemed sensitive.^56^ Concerned about the proliferation of unauthorised copying and piracy, the imperial Chinese state crafted various penalties to restrict such unauthorised reproductions.^57^ Those who sought to make unauthorised use or reproduction of works (that were not under exclusive state control) were subject to 100 lashes with a heavy bamboo cane and the destruction of the reproduced items.^58^ Persons who reproduced state-controlled items risked more severe punishment. According to the *Penal Conspectus*, ‘Those who reproduce state-controlled items, spread the items …, and transmit them to stir up the multitude …, shall get a death sentence by strangulation’.^59^ This high degree of state control and penalty over the unauthorised reproduction of items was established in order to sustain imperial power. This highlights the fact that while the 15th century witnessed the emergence of a mechanism towards an individualised form of IP in Europe, no indigenous counterparts were evolving in China. On the contrary, the state endeavoured to regulate this issue by predominantly focusing on how best to maintain its sovereign honour and authority.

Throughout the imperial history of China (until 1912), there were no formal or informal sources of laws which vested individuals, communities, or families seeking to maintain their monopoly over intellectual creations with ‘rights’ that could be enforced against the imperial state or others.^60^ Almost all known cases of state support, for what we now call IP, in imperial China were directed largely towards the acquisition and maintenance of imperial power.^61^ Central here was the decision as to which knowledge should be allowed for dissemination and which knowledge should be circumscribed in the best interests of the imperial power.^62^

One of the most significant practices associated with China’s imperial power was the Sinocentric approach developed by the imperial state toward western ideas and knowledge.^63^ The Chinese historically dismissed western foreigners as ‘outer barbarians’ and were sceptical of western science, institutions, and ideas including the concept of IP.^64^ The imperial leaders of China remained fixed in their belief in having superior wisdom and knowledge surpassing that of the ‘outer barbarians’.^65^ The Emperor Qianlong of the Qing dynasty, for example, dismissed King George III of England’s envoy with the famous words, ‘We possess all things. I set no value on objects strange or ingenious, and have no use for your country’s manufactures’.^66^ China’s long closed door was blasted open by western colonialists in the Opium War (1839–42) and subsequent Arrow War (1856–60). After this, and for the first time, the western system of IP rights was imported into China ‘with such inventions and novel ideas as the gunboat, opium, most favored nation trading status, and the extraterritorial system’.^67^

Nevertheless, China’s defeats in the Opium War and the humiliation suffered under various unequal treaties made the Chinese comply selectively with or resist western IP laws and other international instruments altogether.^68^ From a Chinese perspective, the western IP system was viewed as representative of the expansion of western powers and the rising foreign encroachments.^69^ As noted by Hongzhang Li, an influential government official in the period towards the close of imperial China (1850s–1890s), when China adopted international IP agreements and other international treaties, ‘it was under the threat of force’ and the Chinese ‘were threatened and deceived’.^70^ This historical experience and perception of international IP laws and other international treaties as ‘hypocritical’, ‘deceptive,’ and ‘threatening’, are important in understanding why the western IP system has never really taken hold in China.

Foreign pressure, coercive trade policies, and deceptive practices exerted by the western powers have contributed to China’s cultural resistance to IP. In 1898 under foreign pressure and driven by ‘sham’ promises (by foreign imperialists) of ending the unequal treaties, in particular the extraterritoriality provisions, Emperor Guangxu formally issued the Bylaws of Awarding Industrial Inventions, which is considered to be ‘the first modern experiment in intellectual property protection’ in Chinese history.^71^ However, once the government realised that the adoption of western-style IP laws would not put an end to unequal treaties signed in the late 19th century, and would not change China’s semi-colonial status, it lost interest in pursuing the reforms.^72^ Since then, China has become sceptical of IP and regarded it as an extractive device that favoured western ‘knowledge monopolists’.^73^ This resisting and sceptical attitude toward IP persisted until the 1970s.

Before 1978, there were no practical IP-related laws in China.^74^ As noted by Niklas Bruun and Liguo Zhang, the socialist transformation of the economy in China essentially abolished all private and individual ownership and so IP rights were not needed.^75^ In 1963, China’s central government established the Regulation on Invention Reward, under which inventors were not allowed to apply for IP rights but received a lump-sum bonus instead. Article 23 of the Regulation on Invention Reward provided that ‘all the inventions belong to the state, no individuals and organisations are allowed to apply for a monopoly. All the organisations across the nation, including collectively owned corporations can use them’. It thus appears that the Regulation on Invention Reward merely sought to preserve as much discretion as possible for the state. It did not offer any ongoing property rights and interests to the inventors. Instead, the state could determine how the inventions could thereafter be used by other third parties, without the prior consent of inventors or the payment of a licensing fee.

With the radical economic and political reforms that occurred at the end of 1978, the legal system in China slowly began to converge with western legal traditions.^76^ This situation was important in the development of IP-related laws in China. It contrasts with China’s different attitude to the western notion of IP rights in the early decades of the 20th century.^77^ The IP regime, which appeared in the early years of the new China, rested mainly on the ideas that individual achievements and accomplishments belonged to all of society.^78^ But since 1978 the Chinese leadership realised that foreign investors would be more willing to invest in China if the nation protected investors’ individual rights, particularly in the area of IP.^79^ As a result, the country took the first steps toward establishing a comprehensive IP regime, that often showed a striking resemblance to those in western countries.^80^ In particular, the Chinese government made great efforts to promulgate new patent, copyright, and trademark laws, as well as facilitating China’s accession to international IP treaty regimes.^81^ These efforts have encouraged overseas enterprises to part with their technology and investment, and channel the desired capital to stimulate China’s socialist modernisation.^82^ Nevertheless, since these IP-related laws were first adopted, the enforcement of IP rights had been ineffective and non-deterrent, which motivated western countries to take actions targeting China’s ineffective IP enforcement.^83^

Since the 1980s, exogenous pressure, particularly from the USA, has contributed to the development of IP policies and laws in China.^84^ Under this external pressure, China has taken a series of measures to bring its IP laws into greater alignment with the expectations of the US government.^85^ China has promulgated new patent, copyright, and trademark laws, as well as facilitating its accession to international IP treaty regimes.^86^ However, despite these developments, many criticisms persist concerning the IP regime in China.^87^ The focus of these criticisms has turned to IP enforcement mechanisms that exist to enforce the substantive rights and duties created by the legislation.^88^ It is normally concluded that ‘protection for intellectual property in China remains closer to rhetoric than reality’.^89^ This is because the cultural character and differing historical experiences of China and its people make the enforcement of IP rights within this jurisdiction challenging, if not futile.^90^ Therefore, without practically considering the difficulties in integrating western legal values in China’s historical and socio-cultural context, adopting western-centric IP laws can arguably be seen to be superficial or ‘in name only’.

Chinese culture, traditional values, and historical background are arguably the main barriers to the growth of effective IP protection in China.^91^ TMK, as I explained in Section I, can be seen as the embodiment of the culture, worldviews, and traditional values of China. Thus, generally, western constructs of IP rights are somewhat alien to TMK (particularly the cultural identity and traditional values embodied in TMK) in China. Perhaps more appropriately, they do not fit squarely into them.

A critical issue concerning the relationships between IP law and TMK is their interactions with western science. As Chidi Oguamanam has stated, ‘western IP rights legitimize a narrow view of science. Nevertheless, it does not acknowledge a different cultural account of knowledge formation’.^92^ For example, western IP laws do not recognise the collective rights over TMK, which prevail within local communities and traditional societies. As argued by many western scholars, western IP laws are mainly based on western scientific paradigms in which all other types of knowledge and theories of knowledge should be either delegitimised or assimilated to bring them into alignment with the episteme of western culture.^93^ More significantly, the arrival of westerners in China came with the assumptions that no frameworks existed in China for regulating and protecting IP.^94^ As a result, western scientific knowledge was compared with TMK in China with a conclusion that TMK was crude, inferior, superstitious and not worthy of preserving, while western scientific knowledge was the essence of the modern world and thus deserving protection.^95^

This situation reflects what Jane Anderson has contended, that the concept of IP merely promotes western cultural interpretations of knowledge, ownership, individual property, and monopoly power.^96^ This means that Chinese ways of interpreting and understanding their TMK systems and knowledge practices are either delegitimised or sidelined.^97^

## III. INTERNATIONAL IP LAW AND THE PROTECTION OF TMK: THE CHINESE APPROACH

### A. Overview of the international framework for IP

The international IP system includes a variety of interactions and linkages among international treaties, international organisations, and plurilateral and bilateral negotiating venues.^98^ As one of the most influential international IP lawmaking venues and treaty-based organisations, the WIPO has housed various IP conventions that form the cornerstones upon which almost all existing international IP regimes and instruments are built.^99^ Two major treaties that the WIPO administers are the Paris Convention for the Protection of Industrial Property and the Berne Convention for the Protection of Literary and Artistic Works. These Conventions are based on two principles.^100^ First, Member States must provide in their national law certain minimum standards of IP protection, namely *substantive minima*.^101^ Secondly, Member States are required to give the nationals of other Member States the same IP protection as their own nationals.^102^ With this foundation, the conventions provided for the protection for copyrights, patents, trademarks, industrial designs, utility models, service marks, trade names, geographical indications, and the repression of unfair competition. The WIPO Convention, adopted in Stockholm in 1967, is also important as it was the WIPO’s founding convention. It represented the beginnings of the first international organisation focusing solely on IP.^103^ According to Article 3(i) of the WIPO Convention, the main aim of the WIPO is to ‘promote the protection of IP throughout the world through cooperation among States and, where appropriate, in collaboration with any other international organization’. Its mission is ‘to lead the development of a balanced and effective international IP system that enables innovation and creativity for the benefit of all’. Despite this, over the past six decades, there has been little improvement in the norms of IP in the WIPO.^104^ Suggested reasons for this include the WIPO’s lack of cross-sectoral negotiating ability and failure to provide effective protection and enforcement mechanisms for IP.^105^ These factors led some technologically advanced countries to push for negotiations on IP by the WTO, leading to the adoption of the TRIPs Agreement.^106^ The TRIPs Agreement is a core component of WTO-administered agreements, which has incorporated a series of WIPO-administered treaties (including the Berne and Paris Conventions).

The TRIPs expanded the protection to all types of IP rights, including trademarks, patents, copyrights, industrial designs, trade secrets, geographical indicators, integrated circuit industrial designs, and other related IP rights.^107^ The aims of the TRIPs are to narrow the gaps between how IP rights are governed in different states and, in this way, to promote international trade.^108^ It also establishes the universal minimum standards for all aspects of the protection and enforcement of IP rights, to fill the gaps that are not covered by national IP laws.^109^ Most significantly, unlike WIPO-administered treaties, the TRIPs has the power to sanction treaty violations through a dispute settlement body.^110^ Consequently, it may be argued that the TRIPs provides Member States of the WTO with the maximum possible security and predictability, and makes IPR protection an integral part of the WTO trading regime.

The TRIPs has established minimum standards for the regulations of various forms of IP. Although the WTO’s Member States cannot provide a level of protection lower than the one established by these minimum standards, they are allowed to incorporate higher levels of IP protection as long as the basic principles of the most favoured nation and national treatment under TRIPs are applied. However, if a country is forced to implement higher levels and more extensive standards of IP protection than what is required by the TRIPs, it can be said that so-called ‘TRIPs-plus’ standards have been implemented by this country.^111^ In this regard, then, the TRIPs is not the end of the story. New developments have occurred at the regional and bilateral level that build further on such standards.

Many technologically advanced countries have chosen to use their unequal bargaining power to reach bilateral or regional trade agreements with low and middle-income countries, thus imposing TRIPs-plus obligations through a country-by-country approach.^112^ For instance, the US’ pursuit of TRIPs-plus protection for medicines in bilateral and regional trade agreements is well recognised.^113^ Until 2021, the USA had achieved bilateral or regional trade agreements with 20 countries, all of which included TRIPs-plus provisions.^114^ The most prominent TRIPs-plus provisions comprise patent term extensions, data exclusivity, and patent linkages.^115^ This effort is believed to be motivated by an intention to achieve the desired levels of protection expected from TRIPs but which TRIPs itself is unable to secure.^116^ Consequently, stronger IP protection is considered more easily secured through bilateral or regional agreements than in the trade agreements within the WTO regime.^117^

### B. China’s engagement with the international, regional, and bilateral IP agreements

From the second half of the 20th century to the present, technologically advanced countries have made great efforts to standardise and expand IP rights across the globe. In this regard, China has often found itself having to enact laws and policies to comply with the changing standards mandated by various international, regional, and bilateral arrangements and agreements related to IP. This situation has led to substantial changes in China’s IP systems.^118^ Take for instance China’s patent system. China has enacted and amended its patent laws no fewer than five times in a span of 36 years (in 1984, 1992, 2000, 2009, and 2020), in order to bring its national legislation into closer compliance with international, regional, and bilateral IP agreements. ^119^

China’s earliest international IP engagement can be traced back to China’s accession to the WIPO in the 1980s. For the first time, this action aligned China with internationally accepted standards of IP protection and helped create its embryonic form of the modern IP system. China adopted its first patent law in 1984,^120^ which included many features similar to the established patent laws in technologically advanced countries. To be specific, the internationally recognised criteria of ‘novelty, non-obviousness and utility’ were adopted to determine whether an invention is patent-eligible.^121^ Similarly to the ‘written description’ and ‘enablement’ requirements of USA and other market economy countries’ practice, this Chinese patent law (1984) provided that ‘the claims shall be supported by the description’ and ‘the description shall set forth the invention in a manner sufficiently clear and complete so as to enable a person skilled in the relevant field of technology to carry it out’.^122^ Similarly to the systems of European countries and Japan, China’s patent system was based on the first-to-file standard rather than the first-to-use standard.^123^ To align with Article 27(2) and (3) of the TRIPs, patent-eligible subject matter excludes scientific discoveries, methods for intellectual activities, diagnostic and therapeutic methods for the treatment of diseases, animal and plant varieties, and new materials created through nuclear reaction.^124^ Significantly, the Chinese patent law (1984) also defined the scope of the monopoly obtained as a result of the patent grant, which conferred upon a patent holder the exclusive right in making, using or selling the patented invention.^125^

Nevertheless, despite its ground-breaking nature, the new patent law was criticised by western scholars. Laurence P Harrington, for example, described it as ‘the emperor’s new clothes’ because of the structural defects and ambiguities embedded in the law.^126^ Major concerns were related to the patent subject matter limitations. The Chinese patent law (1984) limited the patentability of chemical products and pharmaceuticals because only processes, rather than products themselves, could receive patents.^127^ Other defects included process problems, enormous powers retained by the state, and difficulties of enforcement.^128^ Indeed, complaints by foreign patent holders of counterfeiting and infringement conducted by the Chinese continued throughout the 1980s.^129^

This situation led the US Trade Representative to institute a section 301 investigation (based on the Trade Act of 1974) against China’s alleged insufficient protection of IP rights.^130^ A section 301 investigation is a mechanism designed to grant the US executive a range of authorities to investigate the violation of trade practices and protect US economic interests abroad under trade agreements.^131^ Prior to 1995 and the initiation of the WTO, the USA extensively utilized Section 301 as a tool to pressure other countries to modify their laws and practices to provide more effective protection for IP rights and eliminate trade barriers to the USA.^132^ Initially, it seemed that the Section 301 investigation against China was successful.^133^ Under foreign pressure and threats of sanctions, China reached a Memorandum of Understanding (MOU) on the Protection of IP with the USA, and promised to reform its weak IP rights enforcement system and eliminate its trade barriers to US products.^134^ This MOU was the first bilateral IP agreement signed by China, which required China to tighten up its patent law (1984).^135^ As a result, China adopted the 1992 amendment of this law, with a possible exception for the revised provision for compulsory licences.^136^ This included:

- extending the duration of an invention patent to 20 years from 15 years,^137^ the same term as in the USA (consistent with Article 1(c) of MOU).
- expanding the scope of patentable subject matter to all fields of technology including pharmaceutical and chemical processes and products, for which only process patents were granted previously (consistent with Article 1(a) of MOU).^138^
- allowing the patent holders the rights to prevent the unauthorised sale or importation of patented products, which made the provision of China’s Patent Law on ‘rights conferred’ consistent with Article 1(b) of MOU.^139^

Furthermore, in 2000 as part of its accession package to the WTO, China undertook a significant overhaul of its patent laws.^140^ These efforts were clearly made in direct response to the obligations required by the TRIPs.^141^ For instance, the amendments relating to the enforcement of patent rights, including allowing for criminal liabilities and injunctions, were introduced to comply with Article 50 of the TRIPs.^142^ This revision also extended the rights conferred to include the right to prohibit unauthorised ‘offering for sale’, consistent with Article 28 of the TRIPs.^143^ More revisions regarding the judicial review of patent invalidations and stricter standards for issuing a compulsory licence were also added, in order to comply with Articles 32 and 31 of the TRIPs, respectively. The amendment law established new standards to compute statutory damages,^144^ which made the provision of China’s patent law on statutory damages more consistent with Article 45 of the TRIPs. Nevertheless, despite this significant progress, concerns regarding the extent to which China’s IP laws aligned with the international agreement still remained. For instance, the amended patent law disallowed the right for a granted patent that was contrary to social morality and public interest or violated China’s laws.^145^ Some argued that this situation might help China to justify the exclusion of an invention that would otherwise enjoy patent rights under the TRIPs.^146^

A fear of losing foreign investment if China did not further improve its IP system led to the third amendment of Chinese patent law, which was approved on December 27 2008, and will come into effect on October 1 2009.^147^ This provided for detailed information about interim measures to prevent patent infringement, which included pre-trial evidence preservation and property preservation measures consistent with Article 50 of the TRIPs.^148^ To comply with Article 62(5) of the TRIPs, the third amendment also permitted disappointed patent applicants to appeal against adverse administrative decisions in court.^149^ The amendment raised the novelty criteria to an ‘absolute novelty standard’, which means that a patent application must be filed prior to any public disclosure.^150^ Such recognition of any public disclosure for contesting the novelty of a subsequent application was consistent with the spirit of the TRIPs, which prevented discrimination as to the country origin of the invention in Article 27 of the TRIPs.

Moreover, since 2001 the Doha Declaration on the TRIPs Agreement and Public Health and the decisions made thereafter, initiated the process to help countries with inadequate manufacturing capacities in the pharmaceutical industry to make efficient use of compulsory licensing under Article 31 of the TRIPs.^151^ To adapt to the changes introduced by the Doha Declaration, the third amendment of the Chinese patent law allowed for compulsory licences to manufacture medicine.^152^ Based on the quantity and quality of amendments, it is clear that China has made substantial efforts to comply with the TRIPs and other international agreements.

And yet, despite this significant progress, China was still criticised for providing insufficient protection and enforcement of IP rights, and for the lack of a deterrent effect on infringing activity under the existing IP provisions.^153^ The most intense criticism came from the USA. As noted by the White House Office Trade and Manufacturing Policy, China’s ‘economic aggression’, as the USA understood it, led to many unsolved problems for the international IP system.^154^ Moreover, the US Trade Representative Special 301 Report annually identifies countries that fail to adequately protect IP rights or provide market access to US companies, and has highlighted China’s non-compliance with TRIPs in deterring IP infringement and enforcing IP rights.^155^ Among the identified unsolved issues were forced technology transfer, discriminatory licensing practices, State-backed outbound acquisition of IP and technologies, and IP theft by computer hacking.^156^ Based on the Special 301 Report, the USA filed a complaint to the WTO against China on TRIPs non-compliance and imposed retaliatory trade tariffs on China’s imports in 2018.^157^ The complaint claimed that ‘China deprived foreign IP rights holders of the ability to protect their IP rights in China’.^158^ Under this pressure, China eventually reached the United States–China Economic and Trade Agreement in 2019, known as the Phase One Agreement. The Phase One Agreement contained WTO-plus obligations in IP and other fields, which went beyond China’s WTO accession commitments and criteria of IP protection found in the TRIPs.^159^

After signing this Agreement, China promptly adopted the fourth amendment to the patent law in 2020,^160^ hoping that this would fulfil some of the commitments required by the Phase One Agreement and partially aligning China’s patent systems to those in the USA. For instance, in accordance with Article 1.12 of the Phase One Agreement, China is required to permit patent term extensions to compensate for ‘unreasonable delays’ in the Patent Office and regulatory delay of pharmaceuticals. This commitment was implemented under Article 42 of the fourth and latest amendment to the patent law. More significantly, the fourth amendment provided a patent linkage system for the early resolution of patent infringement disputes prior to competitor pharmaceuticals being potentially launched in China. Such a patent linkage system is consistent with China’s obligations under the Phase One Agreement, and drew from the US Hatch-Waxman Act for resolving patent disputes surrounding generic drugs. Therefore, despite the argument that external pressure is less likely to result in long-term outcomes in China, it is still crucial to acknowledge that it was in response to external pressure from the technologically advanced countries and pressure to comply with international, regional, and bilateral IP agreements that China made substantial changes for its IP system.

Nevertheless, in much of the existing literature it is considered that the international, regional and bilateral IP agreements were largely established to cater to the needs of the technologically advanced countries’ economies.^161^ It is further argued that such agreements largely ignored the extent to which China could affordably build stronger IP protection and, in return, gain greater access to the global value chain dominated by these technologically advanced countries. In this regard, as noted by some scholars, these agreements might be consistent with the interests of the technologically advanced countries, but might incur greater costs than benefits when applied in China.^162^ Moreover, the bilateral agreements (such as the USA–China Phase One Agreement) that pressure China to adopt a higher level of IP protection norms could undermine the ‘policy space’ and ‘balance’ permitted in the TRIPs and other multilateral treaty frameworks, thus restricting China’s options and uses of flexibilities under the TRIPs.^163^

Therefore, it can be argued that the foreign efforts to bring China’s IP practices up to international standards through pressure and resulting agreements may add symbolic value and normative rhetoric to the legal and policy changes, but runs counter to the practical reality in China. This is because the Chinese culture lacks in acceptance of, and in many cases resists the notion of, IP rights.^164^ External pressure would not lead to the concepts of IP rights being incorporated into the consciousness and culture of Chinese people, but enhance their resistance and hostility towards IP rights. Just as Ke Shao has described, the technologically advanced countries and many international organisations merely play a role akin to that of a Christian missionary, evangelising the Chinese with the western IP perspective which is skewed towards the interests of powerful industries and companies in technologically advanced countries.^165^

In doing so, this largely ignores China’s political culture, the intellectual tradition developed through Confucianism, and many other non-legal factors that have shaped Chinese attitudes toward IP rights.^166^ For instance, as William Alford has argued, the concept of IP rights was historically and culturally unable to take root in China because of the tight political control over publication work, and Confucianism’s focus on learning through imitation and reproduction.^167^ This situation has led to the belief that the fundamental principles of democracy and the rules of law that influence western attitudes to IP rights, have no indigenous counterparts in Chinese culture and history.^168^

However, traditionally having no indigenous notion of IP rights in China does not mean that China is entirely unable to build its domestically suitable IP framework under the international, regional, and bilateral IP agreements. The point is that applying a western-style IP system in China is not as simple as adhering to the standards established by the international agreements. For the law to be effective, it must put a specific emphasis on the understanding of the society and the distinctive culture, values, traditions, and customs which characterise the country in which the law is applied.^169^ If this is the case, then Chinese people may change their attitudes and have incentives to utilise the law and require institutions to enforce the law which has been made.

### C. China’s approach towards protecting TMK under international IP agreements

In a variety of international fora, agreements, and policy networks, work has been done on the protection of traditional knowledge (including TMK) under IP law. One typical proposal has been that the IP rights applicants should have an obligation of disclosure relating to the utilisation of traditional knowledge (including TMK) and associated genetic resources.^170^ Some biodiversity-rich countries such as the African Group and the Andean Community, made submissions to the TRIPs Council to seek the implementation of obligations to disclose the origin of traditional knowledge (including TMK) and associated genetic resources as a significant mechanism for reconciling the TRIPs with the Convention on Biological Diversity (CBD).^171^ This legal topic was expressly listed in the agenda of the WTO TRIPs Council at the fourth Ministerial Conference of the WTO in Doha, Qatar, in 2001.^172^ Under the negotiation, at least two possible amendment methods were suggested to the TRIPs, each with its own unique wording. The first method was an amendment to Article 27 of the TRIPs, by adding an exception to patentability which provides that:

Members may also exclude from patentability the products or processes which directly or indirectly include genetic resources or traditional knowledge obtained in the absence of compliance with international and national legislation on the subject.^173^

The other approach is an amendment to Article 29 of the TRIPs by including the following provision:

Members shall require an applicant for a patent to disclose the country and area of origin of any biological resources and traditional knowledge used or involved in the invention, and to provide confirmation of compliance with all access regulations in the country of origin.^174^

Many technologically advanced countries, though, opposed the proposal for the TRIPs amendment, arguing that adding the disclosure requirement in TRIPs would lead to ‘legal uncertainty and other negative consequences’.^175^ Consequently, the negotiation process at the WTO was stuck because of these divergent views.

Developments in the WIPO has, though, contributed to the discussion about disclosure requirements in IP rights applications. The Conference of the Parties of the CBD invited the WIPO to explore the options for model provisions on disclosure requirements in IP rights applications.^176^ The WIPO General Assembly decided that the WIPO should respond positively to this invitation.^177^ Diverse views were considered during debates of the WIPO IGC, and some biodiversity-rich countries submitted proposals on a disclosure of origin obligation, informed by their practical experience in implementing such an obligation in their domestic legislation. For instance, the delegation from China was of the opinion that the introduction of disclosure requirements should be incorporated into the existing IP system, which could help align the IP system with the CBD, as well as facilitating the implementation of prior informed consent and benefit-sharing.^178^ Given that China’s national patent law had established the detailed principles for the disclosure requirements in the patent application, the Chinese Delegation also supported the amendment of WIPO-administered treaties such as the Patent Law Treaty and Patent Cooperation Treaty to include relevant disclosure requirements.^179^ Nevertheless, consensus was blocked by some technologically advanced countries, who basically considered the disclosure requirement as an innovation-deterring burden, which introduced elements of legal uncertainty.^180^ Take, for instance, the statements made during the IGC Session:

Any instrument would need to ensure that patent systems and related IPRs would not be threatened by any element of legal uncertainty … A disclosure requirement which would create legal uncertainty in the patent system would not be in the interest of the Member States and patent users.^181^

Although there was progress in the discussions about disclosure requirements regarding the utilisation of Traditional Knowledge (including TMK) in various international forums, such requirements for the utilization of TMK and associated genetic resources have not been put into effect globally because an agreement or consensus has not been reached among the participating nations.^182^ In this regard, China has the flexibility to choose a suitable model for its national lawmaking. At China’s domestic level, China’s highest legislative body, the National People’s Congress, promulgated the Third Amendment to the Patent Law in 2008, which took effect in 2009.^183^ This amendment introduced a new provision that required patent applicants to declare the origin of genetic resources in a patent application despite the disclosure requirements for TMK not explicitly being mentioned. It provides that:

Concerning an invention accomplished by relying on genetic resources, the applicant shall, in the patent application documents, indicate the origin and direct source of the genetic resources. If the applicant cannot indicate the origin, he/she shall state the reasons.^184^

Nevertheless, some scholars have argued that the disclosure requirement included in Article 26 of China’s amended patent law and patent application procedure was merely a formal requirement, which did not require the Patent Office to check the credibility of the disclosed information.^185^

Such a standalone provision would not effectively prevent the misappropriation of related resources and knowledge. Therefore, China’s amended patent law (2008) included another provision seeking to compensate for the insufficiencies of the weak disclosure mechanism established in Article 26. This provision provided that patentability to any invention relying on genetic resources would be denied if the use of the underlying genetic resources is in violation Chinese law or regulation.^186^ Arguably, this provision would create a strong deterrent on non-compliance because failure to comply could lead to either the rejection or invalidation of a patent.

Against this backdrop, it is fair to say that China’s national patent law has mandated disclosure requirements more extensively than was discussed at the international level, which many developing countries might wish to evaluate and draw upon. As China’s State Intellectual Property Administration has noted, it is ‘in the best interest of China to align with the same practice of developing countries in a field where international treaties have always focused on the interest of developed countries’.^187^

The WIPO has also initiated efforts to explore forms of IP protection for TK by forming the WIPO Fact-finding Missions (WIPO-FFMs) and the WIPO IGC. As part of these efforts, the WIPO IGC commissioned a gap analysis, ^188^ which identified four critical gaps in the existing legislation on the protection of traditional knowledge: (i) such knowledge is not an eligible subject matter for IP protection under existing IP law; (ii) existing IP systems do not recognise collective or community ownership, and so exclude indigenous and local communities from the benefits of IP protection; (iii) some critical forms of protection, such as the specific disclosure requirement relating TK, have not been provided under existing IP international standards; and (iv) the entitlement to remuneration from the use of traditional knowledge is absent.^189^

To address these gaps, certain options were suggested that either exist or might be developed.^190^ A number of adjustments to the application of existing IP rights and potential new (sui generis) adjuncts were then proposed.^191^ The proposed adaptations are mainly characterised as ‘defensive’ or ‘positive’.^192^ Defensive protection aims to establish a mechanism that prevents third parties who are outside of the communities from obtaining IP rights over TK. There are two options for defensive protection: first, adopting the legal, administrative, and judicial measures necessary that recognise TK as prior art relating to patentability; secondly, creating a database to make TK searchable by patent offices. Positive protection is oriented to grant exclusive IP rights in TK, which aims to empower communities to assert their indigenous rights to ownership, control their TK, and allow them to take actions against the misappropriation of their TK. The options of positive protection are usually reflected in two types of legal remedies: making effective use of conventional IP laws, and establishing ‘sui generis’ or special laws to address the positive protection of TK. These are complementary strategies that should be regarded as balancing elements that are essential for the realisation of comprehensive protection of TK. As a longer term approach, there have been many initiatives and calls from developing countries for a binding international sui generis system for providing comprehensive protection of TK.

At the national level, China did not choose between the two options. It followed the high-biodiversity countries’ approaches to combine them flexibly to suit its own needs, priorities, and preferences. First, a defensive protection measure has been implemented in China by establishing a series of TMK databases.^193^ The largest TMK database is set up by the Institute of Traditional Chinese Medicine Information (the Traditional Chinese Medicine Database), which includes around 48 categories of sub-databases, possessing 120,000 items relating to China’s TMK.^194^ In addition, the China Traditional Chinese Medicine Patent Database is produced and maintained by the State Intellectual Property Office which includes more than 22,000 of China’s TMK related patent records.^195^

Arguably, despite being a non-legal mechanism, these databases perform the significant functions of safeguarding TMK in China. This is because the compilation of TMK in a digital format can be used as evidence of prior art by the patent examiners and other authorities in China to prevent third parties from erroneously obtaining patents derived from TMK. More importantly, this defensive protection measure is consistent with China’s distinctive linguistic and cultural constructs. Unlike many other multi-ethnic states with a great variety of languages, in its recent history China successfully standardised and unified its language system to smooth out multiple discourses and the process of communication across the country.^196^ On this basis, all official documents, deeds, and communications (including TMK documents and databases) in China recognise Mandarin as a common official language.^197^ Therefore, while the TMK document’s original language is still available in the database and represents the authentic text, the use of one single official language for standardised translation could make the database more accessible. It allows users to search for any information regarding TMK across the entire database, regardless of the original language. More significantly, the single language format in the TMK database eliminates the need for the Patent Office to recruit and train additional examiners with expertise in multiple ethnic languages. Ostensibly, it simplifies the patent application and examination process and significantly reduces the confusion over multi-ethnic filing practices.

As China’s policies are increasingly geared towards promoting the integration of its TMK into modern western biomedical practices and facilitating innovation in TMK, China has embraced its existing patent system as a viable positive protection mechanism for TMK.^198^ This is because TMK that has incorporated western scientific techniques would make the determination of patentability more straightforward and predictable. Meanwhile, the Chinese government is also pushing for proactive measures towards the recognition and maintenance of TMK’s cultural philosophical roots and heritage from the past and nature. According to the Chinese State Council’s Several Opinions on Supporting and Promoting the Development of TMK, the protection and promotion of TMK should ‘adhere to the unity of inheritance and innovation, maintain the characteristic advantages of TMK, and actively apply modern science and technology’.^199^

Arguably, this situation reflects China’s dialectical attitudes towards its positive protection measures in the context of TMK. On one hand, it conveys messages of aggressively integrating its TMK system into the western medical ontology and conventional IP system. On the other hand, it shows a determination to maintain TMK’s natural philosophy and deep theoretical roots which arise from its indigenous practices as an alternative to western biomedicine. Such dialectical approaches are crucial in understanding China’s TMK as a phenomenon emerging from contradictory social interactions in local communities and developing by consistently incorporating new knowledge and therapeutic ideas in response to changing needs in the local environment.^200^

## IV. THE MISMATCH BETWEEN THE WESTERN SYSTEM OF IP RIGHTS AND THE TMK OF CHINA

### A. The conflicts between IP rights and TMK as national cultural heritage: when the confucian paradigm meets western IP theory and practice

In a well-known article, Alexander Eckstein and others considered China’s failure to achieve industrialisation and modern economic growth in the 19th century as a ‘cultural problem’.^201^ It was maintained that the Confucian world views of China as ‘central, superior, and self-sufficient’ were obstacles or barriers that, in the long term, prevented China from embracing capitalism, individualism, and the global market economies manifested by the western philosophical tradition.^202^ Arguably, similar problems also arose within the broader socio-cultural context in which the concepts of IP rights as opposed to TMK exist.

At the national level, China’s TMK embodies its traditional beliefs, values, and practices and reflects Chinese thinking about illness and health.^203^ According to a number of scholars, the Confucian philosophy forms the unique cultural and social context in which TMK has been developing in China.^204^ For instance, ‘*ren*’ (humaneness, benevolence, or human-heartedness) is one of the most fundamental concepts in Confucianism. It arises from the complicated communal and familial system of ancient China.^205^ According to Confucian philosophers, ‘*ren*’ (仁) reflects the ideal world of Confucianism, which considers compassion and empathy as a starting point and seeks to transform moral qualities (compassion and empathy) into virtues that prompt people to behave benevolently, constantly, and consistently.^206^

As noted by Ann Pang White, the ideas behind ‘*ren*’ in Confucian terms have social meaning: seeking to make structural changes that would benefit the less fortunate population, including women, the elderly and the poor.^207^ In this regard, Confucian principles such as ‘*ren*’ are believed to have a strong influence upon the development of TMK in China. In ancient China, people learned medicine and became TMK practitioners, mostly because of the Confucian conviction of their moral obligations to the community, family, or society as a whole.^208^ According to influential Confucian classics (《朱子遗书》Posthumous Papers of Zhu Xi), people with the virtue that Confucius stressed should never leave their ailing parents or relatives in the hands of a quack doctor; this would be considered a violation of Confucian principles. Therefore, it was considered to be the children’s duty to learn medicine (病卧于床, 委之庸医, 比于不慈不孝。事亲者, 亦不可不知医).

Moreover, in an ancient treatise entitled ‘On the Absolute Sincerity of Great Physicians’ (also known as the Chinese Hippocratic Oath), physicians were required to develop a sense of compassion and empathy in the first place, commit to making every effort to save every living creature, treat every patient equally, and not seek wealth while treating patients.^209^ This situation is consistent with the theory of Confucianism. Therefore, just as many Confucian scholars noted, the principles of Confucianism and the TMK of China are inseparable.^210^ Indeed, ‘Medicine is nothing but the application of Confucianism in a healing field’.^211^

According to the Confucian worldview, a clan or family forms the fundamental unit of human community.^212^ TMK is considered as an accomplishment generated from that basic unit, and is expected to be shared.^213^ Being influenced by Confucian thought, in China, creative and non-creative knowledge, including TMK, are generally seen as belonging to the clan or family.^214^ Ownerships over that work or knowledge tends to reflect collective rather than individual needs. This is because, according to the idea of Confucianism, collective or group rights should be placed above individual interests and all types of knowledge and innovation should be for the collective.^215^

In contrast, the concept of IP rights built by technologically advanced countries is considered to be more individualistic,^216^ which is largely alien to Chinese culture and Confucian belief. According to Marci Hamilton, western-style IP law values individual creative endeavours, cherishes original innovation, and chooses the creative individual for recognition and rewards.^217^ For instance, the TRIPs is modelled on western IP practices,^218^ which relies heavily on notions of individuality, exclusive rights, private reward, and imposes them upon countries like China. The preamble of the TRIPs explicitly recognises that IP rights are ‘private rights’. These ideas are fundamentally at odds with China’s deeply held Confucian cultural values and incompatible with the TMK arising from such cultural and social underpinnings of China that values connection with the ancestors and collective identities.

One of the most prevailing justifications for IP rights, at least in western countries and international treaties, is utilitarian theory.^219^ This theory is rooted in the economic understanding of rights which provides that the grant of IP protection could create the commercial incentives of exclusive rights for a limited duration to inventors and helps encourage them to create further innovation.^220^ By recognising IP rights, many inventors could recoup their investment in the creation of the invention, which would yield greater utility and monetary effect on the whole society.^221^

This traditional theoretical justification for IP rights cannot, however, justify the IP protection of TMK in China. This is because cultural tendencies have caused the Chinese to refrain from commercialising and commodifying TMK. These cultural tendencies primarily arise from the influence and impacts of Confucian values (upon which Chinese culture has been based) which stress social commitments rather than individual material gain.^222^ To be precise, Chinese traditional culture has a strong disdain for commercial activity and repudiates the creation and use of knowledge (including TMK) for profit-making.^223^ Confucius even expressed a negative attitude towards profit-making in his Analects, ‘The noble-minded man comprehends righteousness while the inferior man comprehends profit’.^224^ Therefore, it is no surprise that merchants who trade in commodities for personal profits are traditionally considered to be at the bottom of the social status scale in China.^225^ Arguably, these deep-rooted cultural values and traditions are incoherent for those who regard the attaining of personal rewards and benefits as a critical prerequisite to the development of IP rights. Western IP rights which aim to promote the commodification or marketisation of knowledge are thus in tension with TMK that has grown from the distinctive cultural and historical soil of China.

Recognising and protecting innovation and creativity (through IP rights) can largely be seen as a contemporary phenomenon, and as a result of ‘urbanization’ and ‘industrialization’.^226^ In the west, it is common to dismiss knowledge embedded in tradition as being unrelated to the contemporary situation by portraying it as ‘traditional knowledge’.^227^ Such knowledge obtained through tradition or anecdote may be assumed to play no role in shaping and impacting the contemporary world. More significantly, as noted by Christopher Ford, westerners tend to believe that real progress can only be achieved by breaking free of the ancient restraints of tradition.^228^ Whether this is true or not, there are significant differences between western and Chinese attitudes towards the knowledge gained through tradition. In Chinese civilisation, knowledge (including TMK) was traditionally considered as coming from divine inspiration or the past. As Alford stated, ‘The power of the past and its consequences for possession of the fruits of intellectual endeavor’ is impactful throughout Chinese history.^229^ ‘The power of the past’ can be reflected in Confucius’ famous dictums, as quoted by Alford, such as ‘I transmit rather than create; I believe in and love the ancients’ and ‘only through encountering the past would unique insight be provided into the essence of one’s own character’.^230^ These dictums have often been quoted to justify the appropriation of pre-existing knowledge with no need for consent or consultation. In this regard, in China knowledge is usually considered as something to be recovered or mimicked rather than discovered or created, thus disparaging the role of creativity and innovation in the advancement of society. The Chinese literary anecdote ‘Luoyang zhi gui’ (洛阳纸贵) reflects these cultural beliefs and values. The story is that in ancient China, as pre-existing knowledge or works became popular and famous, scholars would compete to copy them so as to express admiration and appreciation for the author. This practice rendered paper as expensive as jade.^231^ Consequently, the traditional knowledge (including TMK) embedded in Chinese cultural and ethical traditions stands in stark contrast to western legal regimes, where laws have been created for the protection of novel knowledge rather than keeping the age-old knowledge alive.

### B. Clashes between IP rights and TMK as a community intellectual and cultural asset: IP frameworks, local medical knowledge and local communities of China

What makes TMK unique is not only the age of the subject matter but also the community-based context of its transmission and creation. This sets TMK apart from other types of knowledge in general.^232^ At the community level in China, ethnic and local communities with their diverse cultures represent a critical component of Chinese civilisation.^233^ Ethnic and local medical knowledge forms part of these communities’ culture and is created in the communal context. This knowledge contains spiritual and cultural elements tapping into the identity of the respective community.^234^ Each ethnic group has created its own system of medical knowledge such as Tibetan medicine, Uyghur medicine or Mongolian medicine, and these represent special branches of China’s TMK.

In recent years, there has been increasingly widespread commercial appropriation of TMK and customary herbal remedies from local communities of China. To be specific, since China’s Tenth Five-Year Plan (2001–2005) was instituted, China has approved and funded many bioprospecting and ethnobotany research projects.^235^ On this basis, until 2016, around 154 pharmaceutical corporations were established (mainly in the territorial area of ethnic and local communities of China) for the development and production of what has become known as ‘ethnomedicine’. This resulted in a total of seven categories and 906 varieties of ethnomedicine products.^236^ These products were based on many nationally reputable TMK and customary herbal remedies from local communities in China, such as Yunnan Baiyao (Yi medicine), Mengwang (Mongolian ethnomedicine), and Cheezheng (Tibetan medicine).^237^ These situations and others have resulted in increased awareness and efforts to protect the TMK of ethnic and local communities, which has extended to the realm of IP law.^238^ Nevertheless, the TMK of these communities presents many challenges for IP protection and the existing western system of IP rights may be ill-suited for the protection of TMK in China.

In China, the TMK of local communities is not only concerned with skills and technology for the treatment and prevention of diseases. It also represents the integration of physical, mental, social, and spiritual practices as holistic exercises to maintain health and prevent disease in local peoples.^239^ For instance, as noted by Geoffrey Samuel, TMK used in the Tibetan community of China is typically viewed as a ‘holistic health system grounded in a deeply spiritual approach to life’.^240^ Unlike the western biomedical tradition that usually disregards the religious, moral, and spiritual dimensions of illness and healing, Tibetan medical practitioners would hope to treat the patient ‘as a whole person, within a wider spiritual framework, rather than as a mere body with a physical problem’.^241^ Nevertheless, aiming to manage the amorphous character of the TMK of such communities, TMK has frequently been transformed and deployed in a narrow sense to comply with IP models. In this process, the communities are usually discounted as a generalised collective origin of ‘tradition’.^242^

So that IP laws could fit TMK easily into the existing operational mechanism, such as the patent system, TMK refers only to pharmaceutical and ‘scientific discursive forms’ while its religious, cultural, and spiritual dimensions are neglected.^243^ As such, IP protection of TMK would largely separate TMK’s technical components from the cultural and traditional values that establish TMK’s collective ownership. This situation could be harmful to the ethnic and local communities of China, contributing to the undermining of cultural and spiritual aspects of their TMK. The TMK of these communities is dynamic and evolving, usually handed down from generation to generation through an oral tradition rather than in fixed form. This knowledge has specific cultural traits. It is not developed for general public access and viewing. Nevertheless, in modern IP laws, novel knowledge must be disclosed in some way to the public,^244^ and ‘fixed’ in a tangible form such as in a written transcription in order for rights to be acknowledged.^245^ For instance, the ‘enablement’ requirement or ‘sufficiency of disclosure’ requirements introduced in patent law demand that a novel idea is disclosed in sufficient detail and in material forms so that the person skilled in the art could implement that idea.^246^ As Teresa Scassa and Fraser Taylor have argued, the main reason for these requirements is that this fixation and sufficient disclosure could serve an evidentiary purpose to facilitate the assessment of possible infringement, in order to enforce and protect the rights vested in the inventor.^247^ However, the oral nature of the TMK of ethnic and local communities would conflict with these ‘enablement’ and ‘fixation’ requirements.

More significantly, according to Catherine Bell and Caeleigh Schier, IP laws merely recognise those who have committed the act of disclosure and fixation as the IP rights holder, rather than those who have actually created the knowledge.^248^ Therefore, while IP rights may be suitable for the societies in which written transcriptions and documentation are privileged, they are not suitable for the ethnic and local communities of China where knowledge has been developed and preserved through an oral tradition.

The traditional development of the TMK of such communities in China raises another problem for the incompatibility and applicability of IP rights to TMK. The development of TMK in China’s ethnic and local communities is normally based on a collective and incremental trial and error process in order to meet local people’s fundamental needs for living and treatment.^249^ For instance, as suggested by Harilal Madhavan, the innovation of the TMK of some ethnic communities in China is reflected by cumulative improvements in existing medical knowledge or finding novel processes of knowledge production and transmission, mostly developed through empirical experience and verified or reinforced through trial and error.^250^ Arguably, this communal and incremental nature of TMK is one of the main reasons why the western IP system is not compatible with the TMK of such communities. This is because IP frameworks (patent law, in particular) are normally characterised by different perceptions and understandings in creativity and innovation that value substantial and notable technical advances rather than gradual and incremental innovation.^251^ Moreover, as a general requirement in IP law, the applications for IP rights must identify the actual inventor or groups of inventors responsible for the creation of the innovation.^252^ However, the gradual and incremental development of TMK makes it difficult or impossible to identify the actual inventors within the community. By applying the requirements for IP protection, such as originality, entitlement, inventive steps, and novelty, to the TMK of these communities, the majority of TMK innovation could be blocked from IP protection. Therefore, as Johanna Gibson has argued, for the purposes of patentability, the criteria of novelty and non-obviousness are ‘unsuitable and artificial criteria to impose upon traditional knowledge in order to make it assimilable within IP law’.^253^

The Chinese patent law (2020) reflects the characteristics of the western IP system by providing protection for a limited duration. Specifically, the duration of an invention patent under Chinese patent law is generally limited to a maximum of 20 years, similar to Western systems of IP rights. For a design patent, this is 15 years and a utility model’s patent lasts for 10 years from the filing date, irrespective of the intrinsic values of the protected information.^254^ As Mohammed Rafiquzzaman has stated, the application of a finite duration of protection in the western system of IP rights is because the longer the protection periods, the greater the marginal benefits that would be reaped by inventors and the greater the value of the new innovation that would be forthcoming.^255^ On this basis, due to the monopolistic use of the innovation, the longer protection periods would also imply a greater value of the associated deadweight losses.^256^ Therefore, in China, finite periods of IP protection have been established to maintain a balance between promoting innovation and avoiding the negative effects of exclusive rights.

Nevertheless, this oversimplifies the complexity of the relationships between TMK and the ethnic and local communities using it in China. In fact, the interests of such communities in TMK are integral to their identities and continuing cultures, as indicated by TMK’s cross-generational nature.^257^ Thus, the significance of TMK to the community exists in perpetuity, not merely for some fixed term. To accept that TMK should be subject to a limited duration of IP protection would be to misunderstand the role that TMK plays in the ethnic and local communities of China. Moreover, the imposition of finite periods of protection on the TMK of such communities is an affront to these communities’ culture and their ancestors. This indicates that the TMK of ethnic and local communities in China cannot easily be fitted into finite time periods, as advocated by western systems of IP rights.

### C. The barriers imposed by the western requirements for IP protection on TMK in China: the example of the patent system

#### 1. Evidentiary obstacles

The rigorous evidentiary standards posed by the western patent system are TMK’s primary external obstacle in China. One of the typical evidentiary barriers that TMK faces in patent law arises from the concept of ‘prior art’ as embedded in patent law. This refers to any documentation and materials published prior to the application for patent.^258^ In China, there are diverse forms of TMK that are subjected to various levels of public exposure and dissemination.^259^ Some TMK, such as Hani-Akha medicine, has been recorded and transmitted through oral tradition (within local communities) for thousands of years, and is, in most cases, undocumented.^260^ From an evidentiary perspective, there would be difficulties in using such undocumented knowledge as prior art according to the criteria required by most countries' patent regimes. Moreover, in some rare cases where TMK has been documented, this tends to have been recorded in secret terms that might not be understood by patent examiners. It thus fails to be recognised as prior art evidence to bar third parties from patenting the same TMK. A typical example is the medical knowledge documented by the 15th century Tibetan medical practitioner, Desi Sanggyé Gyatso, related to the healing of life-wind illnesses.^261^ In his medical texts, ‘secret’ terms are scattered throughout, which render most of his manuscripts puzzling to people lacking the decrypting key.^262^ Therefore, considering the varieties of China’s TMK, evidentiary barriers exist which impede its protection via IP rights. These occur when asserting that a specific form of TMK satisfies the patentability requirements to support a patent application, or a particular type of TMK is prior art evidence to defend against a third party’s patent application.

#### 2. Substantive obstacles

TMK in China also faces substantive obstacles under the existing patent system, given its enormous variety and unique characteristics. This is mainly because the patentability requirements in Chinese patent law do not recognise the wide variety and distinctions that exist in the development and use of TMK in China. This thus creates obstacles in providing a blanket solution for protecting all forms of TMK in China.

In particular, first, it is a fundamental principle of the Chinese patent regime that an innovation is novel if it is not anticipated by prior art immediately before the filing date of a patent application.^263^ On this basis, as one of the conditions for patentability, the novelty requirement arguably constitutes a substantive barrier for TMK holders to obtain patent protection. This is because a wide array of TMK and practice in China are fixed through being codified or recorded, which is sufficient to destroy the novelty and, hence, the patentability required for patenting TMK.^264^ A typical example of this type of TMK in China is Chinese herbal medicine (Han ethnic medicine (汉族中医)),^265^ which was the most influential traditional medicine in China and has usually been codified in written form in renowned medical classics, which have been widely circulated among Chinese people.^266^ Because of this, it is difficult for such herbal medicine to meet the patentability criteria of novelty required by Chinese patent law.

Aside from the novelty requirement, another substantive barrier arises from the inventiveness threshold for patentability. In the Chinese jurisdiction, an invention is considered to have inventiveness if, having regard to the state of the art, it is not ‘obvious’ to a person skilled in the art.^267^ In assessing this lack of obviousness, differences must be first identified between the claimed invention and the closest prior art.^268^ Such a requirement, however, constitutes an obstacle in applying these inventiveness standards to TMK. This is because TMK in China is often a mix of a dozen or more herbs or ingredients. Even one single herb or ingredient may contain hundreds of unknown compounds, thus making it extremely difficult to identify the differences between the claimed innovation and the prior art.^269^

Furthermore, patent law requires that, after the differences are identified, the problem to be solved, the solution to the problem, and the advantageous effect, if any, of the claimed invention relative to the closest prior art, should be considered (the problem, solution, and effect test).^270^ When applying these tests to China’s TMK, a vast amount of TMK would be barred from access to patent protection because of its incremental characteristic. ‘The problem, solution, and effect tests’ adopted by the Chinese Patent Office suggest that inventiveness can be satisfied if and only if the claimed invention can address an unexpected and prominent solution to the problem, rather than an incremental or predictable solution.^271^ On this basis, the threshold of inventiveness in Chinese patent law leaves no room for any TMK characterised as an incremental improvement over the existing knowledge that aims to meet the fundamental health needs of communities.

Another patentability criterion required by Chinese patent law provides that a claimed invention must be disclosed in sufficient detail to enable the person skilled in the art to understand and carry out the content of the invention.^272^ Such disclosure requirements could present another insurmountable obstacle for patenting TMK in China. This is because some forms of TMK have a secret or sacred significance under the customary law of the respective ethnic and local communities.^273^ Typical examples include the above-mentioned ‘secret’ medicine found across the writings of the Tibetan medical tradition, especially ‘secret medicine’ (gsang sman) in the text of Sangs rgyas rgya mtsho’s Extended Commentary.^274^ The secret or sacred nature of such communities’ TMK represents their cultural and spiritual values and is closely tied to their dignity and self-worth,^275^ which inevitably makes these communities and TMK holders in China less likely to disclose sufficient information about their knowledge to meet the disclosure requirements of the Chinese patent law.

#### 3. Administrative obstacles

The patent system in China also poses administrative challenges that might prove to be daunting for the holders of TMK to overcome in order to obtain patents. For example, first, Article 26 of the Chinese Patent Law provides that the patent application must include the specific name and address of the applicant and the inventor. Identifying a specific inventor of TMK is generally difficult because, as will be clear by now, the ‘ownership’ of most forms of TMK in China is vested in a family group, clan group, or wider community group.^276^ It is therefore hard to prove that one member of the community is the first to invent the knowledge. More significantly, TMK in China is knowledge invented in an incremental fashion from generation to generation.^277^ The incremental development of TMK adds more difficulties in identifying TMK creators in the community, thus it fails to meet the administrative requirements under Chinese patent law.

Secondly, the lack of financial aid and resources is another administrative obstacle in the process of patent application and litigation. In China, the ethnic and local communities that hold TMK, account for a disproportionate percentage of the poor.^278^ According to Lv Feijie, Director of the State Council’s Poverty Alleviation Office, 36.5% of the Chinese population suffering from absolute poverty come from the ethnic and local communities living in the western part of the State, even though China’s ethnic and local communities only make up 8.5% of China’s total population.^279^ The communities holding TMK in China may thus not normally possess the appropriate financial resources to prepare and prosecute the patent applications.

Finally, patent applications are usually written in a painfully convoluted manner, full of scientific jargon.^280^ Given the prevalence of illiteracy and low educational attainment rates in China’s ethnic and local communities,^281^ the more complicated the patent application is, the more administrative difficulties it would create for these communities and the holders of TMK to acquire a patent.

### D. Protection of TMK via tiered or differentiated approaches

The analysis above demonstrates that using an IP system to protect TMK in China’s context is a difficult task. To address this, I propose a tiered approach to the protection of TMK in China, where there are many forms of TMK subjected to various levels of public exposure depending on how these forms are traditionally held, shared, and diffused. This novel approach seeks to delineate various kinds of TMK and identify corresponding rights. This provides a mechanism for two purposes: first, for assessing the types of rights that could be applied to such discrete forms of TMK in China, and, secondly, for determining how IP structures can be reconciled with this mechanism.

In this system, the level of protection is calibrated with regard to different tiers and their extent of diffusion. The tiers can be roughly divided along various patterns in which TMK is held: ‘secret TMK’; ‘TMK closely held by ethnic and local communities’, ‘TMK collectively held by national communities’, and ‘widely diffused TMK that becomes the global stock of knowledge’. On this basis, different kinds of TMK are subject to different levels of rights and obligations in accordance with the degree of their diffusion. In particular, stronger exclusive rights should be granted for the secret TMK, compared with TMK closely held by ethnic and local communities and TMK collectively held by national communities. The TMK widely diffused as the global stock of the knowledge usually attracts the weakest rights claim because it is available at the periphery of the public domain.

The details of this system could be customised in accordance with specific TMK, and the State and communities’ customary protocols and specific national contexts and circumstances. This tiered approach could provide appropriate levels of protection to various forms of TMK and their knowledge holders in China. At the same time, it could provide the ‘user certainty’ and other form of legal confidence to increase TMK users’ awareness of and sensitivity to different rules regarding different forms of TMK. In future research, more clarifications and analysis are needed concerning the specific duties of the different stakeholders associated with the different tiers.

## V. CONCLUSION

The analysis has shown that systems of IP rights sit uneasily with the TMK of China because of China’s unique cultural traits, distinctive national traditions, and the variety and diversity of China’s TMK. From a technical perspective, TMK is simple to duplicate and multiply, and easy to alter and transmit. Therefore, the lack of appropriate protection would have the consequence that TMK could be freely accessed and utilised, while the knowledge providers could not exclude third parties from the use of their knowledge once others have obtained access.

More significantly, the rapid growth in China’s pharmaceutical sector, and its increased engagement in innovation and technology, have challenged the legal system and, in particular, the adaptability of the IP system. This is because the IP rights would frequently lie with the corporation that has created an altered product based on TMK, tweaked some small aspects, and patented it as ‘new’. This situation has raised serious concerns regarding the misappropriation of TMK through IP rights. These concerns necessitate further research which explores the appropriate legal, policy, and administrative measures that are best suited to balance the necessary protection for TMK with the need to preserve access to TMK in China.

## Footnotes

1 Chengguo Zhang, ‘How is the Owner of “Traditional Knowledge Right”? A Perspective of International Law and the Case of China’ (2018) 21 Journal of Legal, Ethical and Regulatory Issues 1.

2 Linda Zhong and others, ‘The Efficacy of Chinese Herbal Medicine as an Adjunctive Therapy for Colorectal Cancer: A Systematic Review and Meta-analysis’ (2012) 20 Complementary Therapies in Medicine 240.

3 Hans Agren, ‘A New Approach to Chinese Traditional Medicine’ (1975) 3 American Journal of Chinese Medicine 207.

4 Huang Hui and Shen Wei, ‘On the Legal Protection for the Security of Intellectual Property Rights of Traditional Chinese Medicine Knowledge’ (2021) 9 China Legal Science 30; Benjamin Liu, ‘Past Cultural Achievement as a Future Technological Resource: Contradictions and Opportunities in the Intellectual Property Protection of Chinese Medicine in China’ (2003) 21 UCLA Pacific Basin Law Journal 75.

5 Fiona Batt, ‘Ancient Indigenous Deoxyribonucleic Acid (DNA) and Intellectual Property Rights’ (2012) 16 The International Journal of Human Rights 152.

6 The 1992 Convention on Biological Diversity (adopted June 5 1992) 1760 UNTS 79, 31 ILM 818 (CBD) art 8(j); Nagoya Protocol on Access to Genetic Resources and the Fair and Equitable Sharing of Benefits Arising from their Utilisation to the Convention on Biological Diversity (adopted 29 October 2010). UNEP/CBD/COP/DEC/X/1 (Nagoya Protocol) arts 5(5), 7 and 12; United Nations Declaration on the Rights of Indigenous Peoples (adopted September 13 1992) A/RES/61/295 (UNDRIP) art 31.

7 G Van Overwalle, ‘Protecting and Sharing Biodiversity and Traditional Knowledge: Holder and User Ttols’ (2005) 53 Ecological Economics 585.

8 Law of the People’s Republic of China on Traditional Chinese Medicine Promulgated by the Standing Committee of National People’s Congress on 25 December 2016, effective from July 1 2017.

9 World Health Organization (WHO), ‘Traditional Medicine Strategy’ <https://apps.who.int/iris/handle/10665/67163> accessed 9 October 2022.

10 Alma Barbaso-Schwartz, ‘Traditional Chinese Medicine: Ancient Holistic Healing’ (2004) 16 Home Health Care Management & Practice 494.

11 He Hong and others, ‘Traditional Naxi Natural Resource Management and Current Policy: A Case Study at Yuhu Village, Yu Long County, Yunnan, China’ in Luchie Maranan (eds), *Indigenous Knowledge and Customary Law in Natural Resource Management: Experiences in Yunnan, China and Haruku, Indonesia* (IWGIA 2010) 1; Mo Gun, ‘The Relationship between Zhuang Folk Traditional Cultural Customs and Zhuang Medicine in China’ (2011) 29 Sichuan Journal of Traditional Chinese Medicine 63; Yang Yang, ‘Preliminary Study on the Connotation of Traditional Chinese Medicine Culture in Yunnan Etiquette and Customs’ (2011) 36 Yunnan Journal of Traditional Chinese Medicine and Materia Medica 1; Xueyan Wang, ‘Evaluation Traditional Chinese Medicine Culture within the Diet Custom of Song Dynasty through the Seasonal Order Poems’ (2019) 28 Chinese Journal of Ethnomedicine and Ethnopharmacy 1.

12 Chidi Oguamanam, ‘Conceptual Perspectives on Biodiversity, Traditional Knowledge, Intellectual Property, and the Protection of Indigenous Peoples in International Law’ in Chidi Oguamanam (ed), *International Law and Indigenous Knowledge* (University of Toronto Press 2006) 58.

13 Krishna Ravi Srinivas, ‘Traditional Knowledge and Intellectual Property Rights: A Note on Issues, Some Solutions and Some Suggestions’ (2008) 3 Asian Journal of WTO & International Health Law and Policy 81.

14 Daniel F Robinson, Pedro Roffe and Ahmed Abdel-Latif, ‘Introduction’ in Ahmed Abdel-Latif and Pedro Roffe Daniel F. Robinson (eds), *Protecting Traditional Knowledge: The WIPO Intergovernmental Committee on Intellectual Property and Genetic Resources, Traditional Knowledge and Folklore* (Routledge 2017) 3.

15 ibid.

16 World Intellectual Property Organization (WIPO), ‘The Protection of Traditional Knowledge: Updated Draft Gap Analysis’ WIPO/GRTKF/IC/37/6, July 20 2018.

17 WIPO, ‘The Protection of Traditional Knowledge’, WIPO/GRTKF/IC/40, June 19 2019; WIPO, ‘The Protection of Traditional Cultural Expression’, WIPO/GRTKF/IC/40, June 19 2019.

18 James D Nason, ‘Traditional Property and Modern Laws: The Need for Native American Community Intellectual Property Rights Legislation’ (2001) 12 Stanford Law and Policy Review 255; Angela Riley, ‘Straight Stealing: Towards an Indigenous System of Cultural Property’ (2005) 80 Washington Law Review 69; Charles R McManis, ‘Fitting Traditional Knowledge Protection and Biopiracy Claims into the Existing Intellectual Property and Unfair Competition Framework’ in Burton Ong (ed), *Intellectual Property and Biological Resources* (Marshall Cavendish Academic 2004) 475.

19 Paul Kuruk, ‘Bridging the Gap Between Traditional Knowledge and Intellectual Property Rights: Is Reciprocity an Answer?' (2004) 7 Journal of World Intellectual Property 429.

20 Thomas Cottier and Marion Panizzon, ‘Legal Perspectives on Traditional Knowledge: The Case for Intellectual Property Protection’ (2004) 7 Journal of International Economic Law 371.

21 Jim Chen, ‘There’s No Such Thing as Biopiracy … and It’s a Good Thing Too’ (2006) 37 Mcgeorge Law Review 1.

22 Owen Dean, ‘Breaking with Tradition: Intellectual Property’ (2013) 13 Without Prejudice 30.

23 Darrell A Posey, ‘Commodification of the Sacred through Intellectual Property Rights’ (2002) 83 Journal of Ethnopharmacology 3.

24 Soo-Myung Choi and others, ‘To Patent or Not to Patent: Case of the Chinese Industry’ (2016) 9(41) Indian Journal of Science and Technology 1.

25 ibid.

26 Adela Teodorescu Calota, ‘Translation as Transplant in Contemporary Law’ (2016) 50 Revista de Stiinte Politice 118.

27 Bichang He, ‘Contemporary Explanation of the Intangible Cultural Heritage of Naxi Dongba Culture’ (International Conference on Art Design, Music and Culture, 2019) 188.

28 Haitao Li and others, ‘Ethnobiological Study on Traditional Medicinal Plants and Fungi Recorded in the Naxi Dongba Sutras’ (2021) 17 Journal of Ethnobiology and Ethnomedicine 1.

29 Yanqiang Zhao and others, ‘Skincare Plants of the Naxi of NW Yunnan, China’ (2020) 42 Plant Diversity 473.

30 Frank D Prager, ‘A History of Intellectual Property from 1545 to 1787’ (1944) 26 Journal of the Patent Office Society 711.

31 Pablo Miguez, ‘Intellectual Property and the Forced Commodification of Knowledge’ (2018) 29 Universitas, Revista de Ciencias Sociales 42.

32 Pamela O Long, ‘Invention, Authorship, Intellectual Property, and the Origin of Patents: Notes Toward a Conceptual History’ (1991) 32 Technology and Culture 846.

33 ibid.

34 Christopher May, ‘The Venetian Moment: New Technologies, Legal Innovation and the Institutional Origins of Intellectual Property’ (2002) 20 Prometheus 159.

35 Joanna Kostylo, ‘From Gunpowder to Print: The Common Origins of Copyright and Patent’ in Ronan Deazley and others (eds), *Privilege and Property Essays on the History of Copyright* (Cambridge OpenBook Publishers 2010) 1.

36 ibid.

37 Linhua Zhang, ‘China’s Traditional Cultural Values and National Identity’ (Carnegie Endowment for International Peace, 2013) <https://carnegieendowment.org/2013/11/21/china-s-traditional-cultural-values-and-national-identity-pub-53613> accessed 9 October 2022.

38 William P Alford, *To Steal a Book Is an Elegant Offense: Intellectual Property Law in Chinese Civilization* (Stanford University Press 1995) 1.

39 ibid.

40 D Greasley and L Oxley, ‘Patenting, Intellectual Property Rights and Sectoral Outputs in Industrial Revolution Britain, 1780–1851’ (2007) 139 Journal of Econometrics 340.

41 Brad Sherman and others, *The Making of Modern Intellectual Property Law: The British Experience, 1760-1911* (CUP 1999) 1.

42 Catherine Seville, ‘The Emergence and Development of Intellectual Property Law in Western Europe’ in Rochelle Dreyfuss and Justine Pila (eds), *The Oxford Handbook of Intellectual Property Law* (OUP 2017) 171.

43 ibid.

44 Natalie Stoianoff, ‘The Development of Intellectual Property Law in China’ in Chan Lai-Ha and others (eds), *China At 60: Global-Local Interactions* (World Scientific 2011) 183.

45 Jia Li, ‘Intangible Assets are More Valuable than the Tangible: Study on the Innovation and Development of the Traditional Time-Honored Brands’ (8th International Conference on Social Science and Education Research, 2018) 242; Liwei Wang, ‘Chinese Traditions Inimical to the Patent Law, The Symposium: Doing Business in China’ (1993) 14(1) Northwestern Journal of International Law & Business 15.

46 ibid.

47 Alford (n 38).

48 ibid.

49 Zhi-fu Qian and Li-li Han, ‘The Winter’s Tale by Shakespeare and the Confucian Values’ (2013) 3 Journal of Literature and Art Studies 333.

50 Linyi Zheng and Xinying Hu, ‘Inventing the Barbarians: The Changing Contexts of Yi in the Late Qing Dynasty’ (2022) 12 Psychology Research 183, 186.

51 Suisheng Zhao, ‘Rethinking the Chinese World Order: The Imperial Cycle and the Rise of China’ (2015) 24 Journal of Contemporary China 961.

52 Zewei Yang, ‘Western International Law and China’s Confucianism in the 19th Century: Collision and Integration’ (2011) 13 Journal of the History of International Law 285.

53 ibid.

54 ibid.

55 Xiangyu Hu, ‘Drawing the Line Between the Civil and the Criminal: A Study of Civil Cases Handled by the Board of Punishment in Qing China’ (2014) 40 Modem China 74; Xue Yang, ‘The Confucianization of Law and the Lenient Punishments in China’ (2015) 10 International Journal of Criminal Justice Sciences 32.

56 Alford (n 38).

57 Sanqiang Qu, ‘A Dilemma for Feudal China: To Launch a Copyright Scheme in the Late 19th Century’ (2010) 5 Frontiers of Law in China 319; Dehui Ye, *Quiet Talks among the Bookstacks* (Shanghai Classics Publishing House 1957) 143.

58 Qu ibid.

59 Hok-Lam Chan, *Control of Publication in China, Past and Present* (The Chinese University of Hong Kong Press 1983) 5.

60 Alford (n 38).

61 Peter K Yu, ‘The Second Coming of Intellectual Property Rights in China’ (2002) 11 Occasional Papers in Intellectual Property from Benjamin N Cardozo School of Law 1.

62 Alford (n 38).

63 Zhao (n 51)

64 JK Fairbank, ‘Tributary Trade and China’s Relations with the West’ (1942) 1 The Far Eastern Quarterly 129.

65 Willian C Johnstone, ‘The Problem of China’ (1970) 14 SAIC Review 21

66 Henrietta Harrison, ‘The Qianlong Emperor’s Letter to George III and the Early-twentieth-century Origins of Ideas about Traditional China’s Foreign Relations’ (2017) 122 American Journal of Ophthalmology 680.

67 Peter Feng, *Intellectual Property in China* (Sweet & Maxwell Asia 2003) 1, 3.

68 Phil Chan, ‘China’s Approaches to International Law since the Opium War’ (2014) Leiden Journal of International Law 859.

69 ibid.

70 S Suzuki, ‘China’s Perceptions of International Society in the Nineteenth Century: Learning More about Power Politics?’ (2004) 28 Asian Perspectives 115, 132.

71 Jian Xu, ‘The History of China’s Patent System and Legislation’ (*China IP Blog*, 2016) <https://niuyie.com/the-history-of-chinas-patent-system-and-legislation/> accessed 19 October 2022.

72 ibid.

73 Servaas Storm, ‘Why the West Grew Rich and the Rest did Not, or How the Present Shapes Our Views of the Past’ (2013) 44(5) Development and Change 1181

74 ibid.

75 Niklas Bruun and Liguo Zhang, ‘Legal Transplant of Intellectual Property Rights in China: Norm Taker or Norm Maker?’ in Niklas Bruun and others (eds), *Governance of Intellectual Property Rights in China and Europe* (Edward Elgar Publishing 2016) 43.

76 Natalie P Stoianoff, ‘The Influence of the WTO over China’s Intellectual Property Regime’ (2012) 34 Sydney Law Review 65.

77 ibid.

78 Sunny Chang, ‘Combating Trademark Squatting in China: New Developments in Chinese Trademark Law and Suggestions for the Future’ (2014) 34 Northwestern Journal of International Law & Business 337.

79 Geoffrey T Willard, ‘An Examination of China’s Emerging Intellectual Property Regime: Historical Uniderpinnings, the Current System and Prospect for the Future’ (1995) 6 International and Comparative Law Review 411.

80 PE Campbell and M Pecht, ‘The Emperor’s New Clothes: Intellectual Property Protections in China’ (2012) 7 Journal of Business and Technology Law 69.

81 Scott J Palmer, ‘An Identity Crisis: Regime Legitimacy and the Politics of Intellectual Property Rights in China’ (2001) 8 Indiana Journal of Global Legal Studies 449.

82 ibid.

83 Donald P Harris, ‘The Honeymoon is Over: the U.S.-China WTO Intellectual Property Complaint’ in Perry Keller (ed), *Law and the Market Economy in China* (Taylor & Francis 2011) 96.

84 Dexin Tian, ‘The USTR Special 301 Reports: An Analysis of the US Hegemonic Pressure Upon the Organizational Change in China’s IPR Regime’ (2008) 1 Chinese Journal of Communication 224.

85 Peter K Yu, ‘Intellectual Property, Economic Development, and the China Puzzle’ in Daniel J Gervais (ed), *Intellectual Property, Trade and Development: Strategies to Optimize Economic Development in a TRIPs Plus Era* (OUP 2007) 173.

86 Palmer (n 81).

87 Thomas Kristie, ‘The Fight Against Piracy: Working within the Administrative Enforcement System in China’ in Hailing Shan, Johan Erauw and Paul Torremans (eds), *Intellectual Property and TRIPS Compliance in China* (Edward Elgar Publishing 2007) 85.

88 ibid.

89 Alexander Hendrie, ‘China’s Commitment to Intellectual Property Rights: More Rhetoric than Reality’ (Property Rights Alliance, October 2014)<http://www.propertyrightsalliance.org/news/china_s_commitment_to_intellectual_property_rights__more_rhetoric_than_reality_-2/> accessed 10 October 2022.

90 Liguo Zhang and Niklas Bruun, ‘Legal Transplantation of Intellectual Property Rights in China: Resistance, Adaptation and Reconciliation’ (2017) 48 *IIC* 4.

91 ibid.

92 Chidi Oguamanam, *International Law and Indigenous Knowledge: Intellectual Property, Plant Biodiversity, and Traditional Medicine* (University of Toronto Press 2006) 158.

93 ibid.

94 Alford (n 38).

95 Yuet-Wah Cheung and others ‘Missionary Doctors vs Chinese Patients: Credibility of Missionary Health Care in Early Twentieth Century China’ (1985) 21 Social Science and Medicine 309.

96 Jane E Anderson, ‘Indigenous/ Traditional knowledge intellectual property’ (2010) Center for the Study of the Public Domain, Duke University School of Law, <https://web.law.duke.edu/cspd/pdf/ip_indigenous-traditionalknowledge.pdf> access 11 October 2022.

97 ibid.

98 Laurence R Helfer, ‘Regime Shifting in the International Intellectual Property System’ (2009) 7 Perspectives on Politics 39.

99 S Bannerman, ‘The World Intellectual Property Organization and the Sustainable Development Agenda’ (2020) 122 Futures 3.

100 Dan Wielsch, ‘Private Governance of Knowledge: Societally-crafted Intellectual Properties Regimes’ (2013) 20 Indian Journal of Global Legal Studies 907.

101 ibid.

102 ibid.

103 Alison Duxbury, ‘The Role of WIPO as an International Organization’ in Sam Ricketson (ed), *Research Handbook on the World Intellectual Property Organization: The First 50 Years and Beyond* (Edward Elgar Publishing 2020) 1.

104 Naomi A Bass, ‘Implications of the TRIPs Agreement for Developing Countries: Pharmaceutical Patent Laws in Brazil and South Africa in the 21st Century’ (2002) 34 The George Washington International Law Review 191.

105 Paul Salmon, ‘Cooperation Between the World Intellectual Property Organization (WIPO) and the World Trade Organization (WTO)’ (2003) 17 Journal of Civil Rights and Economic Development 433.

106 Agreement on Trade-Related Aspects of Intellectual Property Rights, April 15 1994, Marrakesh Agreement Establishing the World Trade Organization, Annex 1C, Legal Instrument Result of The Uruguay Round, 33 I.L.M. 81 (1994) (‘the TRIPs Agreement’).

107 ibid.

108 Stefan Szymanski and Tommaso Valletti, ‘Parallel Trade Price Discrimination, Investment and Price Caps’ (2005) 20 Economic Policy 705; Stephanie Peatman, ‘Moving Toward Uniform International Trademark Protection: How Amending the TRIPS Agreement will Make Parallel Importing of Gray Goods Less Gray’ (2014) 20 Southwestern Journal of International Law 445.

109 Peatman ibid; article 1.1 of TRIPS agreement provides that: ‘Members shall give effect to the provisions of this Agreement. Members may, but shall not be obliged to, implement in their law more extensive protection than is required by this Agreement, provided that such protection does not contravene the provisions of this Agreement. Members shall be free to determine the appropriate method of implementing the provisions of this Agreement within their own legal system and practice’.

110 Laurence R Heifer, ‘Regime Shifting: The TRIPs Agreement and New Dynamics of International Intellectual Property Lawmaking’ (2004) 29 Yale Journal of International Law 1.

111 Peter Drahos, ‘Bits and Bips: Bilateralism in Intellectual Property’ (2001) 4 The Journal of World Intellectual Property 791.

112 Bryan Mercurio, ‘TRIPS-Plus Provisions in FTAs: Recent Trends’ in Lorand Bartels and Federico Ortino’ (eds), *Regional Trade Agreement and the WTO Legal System* (OUP 2006) 215.

113 Ruth Lopert and Deborah Gleeson, ‘The High Price of “free” Trade: U.S. Trade Agreements and Access to Medicines’ (2013) 41 Journal of Law, Medicine & Ethics 199.

114 ‘Trade Agreement’ (Office of United States Trade Representative) <https://ustr.gov/trade-agreements> accessed 11 October 2022.

115 Matthew E Silverman, ‘The Case for Flexible Intellectual Property Protections in the Trans-pacific Partnership’ (2014) 27 Journal of Law and Health 215.

116 ibid; Lopert and Gleeson (n 113).

117 Jean-Frederic Morin, ‘Multilateralizing TRIPs-Plus Agreements: Is the US strategy a failure?' (2009) 12 The Journal of World Intellectual Property 175.

118 Kristie Thomas, ‘Implementing the TRIPS Agreement in China’, Assessing Intellectual Property Compliance in Contemporary China (2017) 85.

119 Peter K Yu, ‘Two Decades of TRIPs in China’ (2021) Texas A&M University School of Law Legal Studies Research Paper No 21-48, 1-12 <http://papers.ssrn.com/sol3/paper.cfm?abstract_id=3965395> accessed 11 October 2022.

120 Patent Law of the People’s Republic of China Amendment promulgated by the Standing Committee of National People’s Congress on March 12 1984.

121 ibid.

122 ibid.

123 ibid.

124 ibid.

125 ibid.

126 Laurence P Harrington, ‘Recent Amendments to China’s Patent Law: The Emperor’s New Clothes’ (1994) 17 Boston College International and Comparative Law Review 337.

127 William E Beaumont, ‘The New Patent Law of the People’s Republic of China (PRC): Evidence of a Second Chinese “renaissance”?’ (1988) 39 IDEA 1; Janiece Marshall, ‘Current Developments in the People’s Republic of China: Has China Changed?’ (1988) 1 Global Business & Development Law Journal 505.

128 Harrington (n 126).

129 Louis S Sorell, ‘A Comparative Analysis of Selected Aspects of Patent Law in China and the United States’ (2002) 11 Washington International Law Journal 319.

130 Trade Act of 1974, 19 USC s 2411 (1974), s 301. s 301 actions allow the U. Trade Representative to impose severe sanctions in the form of greatly increased tariffs on countries found to violate trade practices.

131 Chris Noonan and Victoria Plekhanova, ‘Digital Services Tax: Lessons from the Section 301 Investigation’ (2021) The British Tax Review 83.

132 Krzysztof J Pelc, ‘Constraining Coercion? Legitimacy and its Role in U.S. Trade Policy, 1975 – 2000’ (2010) 64 International Organization 65.

133 Robert B Frost Jr, ‘Intellectual Property Rights Disputes in the 1990s between the People’s Republic of China and the United States’ (1995) 4 Tulane Journal of International and Comparative Law 119.

134 Memorandum of Understanding Between the Government of the People’s Republic of China and the Government of the United States of America on the Protection of Intellectual Property (MOU) (China–United States) (January 17 1992) 34 I.L.M. 676.

135 The provisions related to the proposed China’s Patent Law revisions are included in art 1 of the MOU.

136 Patent Law of the People’s Republic of China Amendment promulgated by the Standing Committee of National People’s Congress on September 4 1992.

137 ibid.

138 Compare 1985 Patent Law, art 25 (excluding ‘pharmaceutical products and substances obtained by means of a chemical process’ from patent protection) with 1992 Patent Law (omitting the exclusion of pharmaceutical products and chemical processes from patent protection).

139 Patent Law (n 136).

140 Patent Law of the People’s Republic of China Amendment promulgated by the Standing Committee of National People’s Congress on August 25 2000.

141 Guangzhou Hu and Gary H Jefferson, ‘A Great Wall of Patents: What is Behind China’s Recent Patent Explosion?’ (2006) 90 Journal of Development Economics 1.

142 Patent Law (n 140).

143 ibid.

144 Patent Law of the People’s Republic of China Amendment promulgated by the Standing Committee of National People’s Congress on December 27 2008.

145 ibid.

146 Veronic Weinstein and Dennis Fernandez, ‘Recent Development in China’s Intellectual Property Laws’ (2004) 3 Chinese Journal of International Law 227.

147 Patent Law (n 144).

148 ibid.

149 ibid.

150 ibid; Guo Shoukang, ‘Some Remarks on the Third Revision Draft of the Chinese Patent Law’ in Martin J Adelman et al (eds), *Patents and Technological Progress in a Globalized World* (Springer Berlin 2009) 713.

151 Matthew Kennedy, ‘When will the Protocol Amending the TRIPS Agreement Enter into Force?’ (2010) 13 Journal of International Economic Law 1; Thomas A Haag, 'TRIPS since Doha: How Far will the WTO Go Toward Modifying the Terms for Compulsory Licensing’ (2002) 84 Journal of the Patent and Trademark Office Society 945.

152 Patent Law (n 144).

153 Konstantina K Athanasakou, ‘China IPR enforcement: Hard as steel or soft as TOFU - Bringing the question to the WTO under TRIPS (2007) 39 Georgetown Journal of International Law 217.

154 White House, ‘How China’s economic aggression threatens the technologies and intellectual property of the United States and the world’ (*White House Office of Trade and Manufacturing Policy*, June 2018) <https://www.whitehouse.gov/wp-content/uploads/2018/06/FINAL-China-Technology-Report-6.18.18-PDF> accessed 14 October 2022.

155 Office of the United States Trade Representative, ‘2018 Special 301 Report’ (2018) <https://ustr.gov/sites/default/files/files/Press/Reports/2018%20Special%20301.pdf> accessed 14 October 2022.

156 Jyh-An Lee, ‘Shifting IP Battlegrounds in the U.S.–China Trade War’ (2020) 43 Columbia Journal of Law & the Arts 147.

157 WTO, ‘China - Certain Measures Concerning the Protection of Intellectual Property Rights Request for Consultation by the United States’, WT/DS542/1 IP/D/38, March 26 2018.

158 ibid.

159 Wang Heng, ‘How to Assess Regional Trade Agreements? Deep FTAs v. China’s Trade Agreements’ (2021) 54 International Lawyer 1.

160 Patent Law of the People’s Republic of China Amendment promulgated by the Standing Committee of the National People’s Congress on October 17 2020.

161 Akalemwa Ngenda, ‘The Nature of the International Intellectual Property System: Universal Norms and Values or Western Chauvinism?’ (2005) 14 Information & Communications Technology Law 59; Christopher May, ‘The Hypocrisy of Forgetfulness: The Contemporary Significance of Early Innovations in Intellectual Property’ (2007) 14 Review of International Political Economy 1.

162 Lisa Forman and others, ‘Addressing Legal and Political Barriers to Global Pharmaceutical Access: Options for Remedying the Impact of the Agreement on Trade-Related Aspects of Intellectual Property Rights (TRIPS) and the Imposition of TRIPS-Plus Standards’ (2008) 3 Health Economics, Policy and Law 229.

163 Ke Shao, ‘What May Validate Intellectual Property in the Traditional Chinese Mind? Examining the U.S.-China IP Disputes through a Historical Inquiry’ (2006) Journal of Information, Law & Technology 1.

164 Liwei Wang, ‘The Chinese Traditions Inimical to the Patent Law’ (1993) 14 Northwestern Journal of International Law & Business 15; Peter K Yu, ‘Intellectual Property and Confucianism’ in Irene Calboli and Srividhya Ragavan (eds), *Diversity in Intellectual Property: Identities, Interests, and Intersections* (CUP 2015) 1.

165 Shao (n 163).

166 ibid

167 Alford (n 38).

168 ibid.

169 Setsuo Miyazawa, ‘Legal Transplants in Contemporary Asia: Foreword’ (2021) 8 Asian Journal of Law and Society 348.

170 Martin A Girsberg, ‘Transparency Measures Under Patent Law Regarding Genetic Resources and Traditional Knowledge: Disclosure of Source and Evidence of Prior Informed Consent and Benefit-sharing’ (2004) 7 The Journal of World Intellectual Property 451.

171 WTO, ‘Draft Modalities for TRIPS Related Issues’, TN/C/W/52, July 19 2008; Daniel Robinson and Margraet Raven, ‘Identifying and Preventing Biopiracy in Australia: Patent Landscapes and Legal Geographies for Plants with Indigenous Australian Uses’ (2017) 48 Australian Geographer 311.

172 WTO, ‘Doha WTO Ministerial 2001: Ministerial Declaration’, WT/MIN (01)/DEC/1, November 14 2001.

173 WTO, ‘Article 27.3(B), Relationship between the TRIPS Agreement and the CBD, and Protection of Traditional Knowledge and Folklore: Communication from Peru’, IP/C/W/447 (June 8 2005).

174 WTO, ‘Taking forward the Review of Article 27.3(b) of the TRIPS Agreement: Joint Communication from the African Group’, IP/C/W/404 (June 26 2003).

175 WTO, ‘Article 27.3(B), Relationship between the TRIPS Agreement and the CBD, and Protection of Traditional Knowledge and Folklore: Communication from the United States’, IP/C/W/469 (March 13 2006).

176 WIPO, ‘Certain Decision of the Seventh Conference of the Parties to the Convention on Biological Diversity’, WIPO/GRTKF/IC/6/13, March 15 2004 <https://www.wipo.int/edocs/mdocs/tk/en/wipo_ grtkf_ic_6/wipo_grtkf_ic_6_13.pdf> accessed 15 October 2022.

177 ibid.

178 WIPO, ‘Draft Report Prepared by the Secretariat’, WIPO/GRTKF/IC/23/8,26 April 2013<https://www.wipo.int/meetings/en/doc_details.jsp?doc_id=241343> accessed 15 October 2022.

179 ibid.

180 C Saez, ‘WIPO Talks on Future of TK, Genetic Resources, Folklore Intensify’ (IP Watch, 2013) <https://www.ip-watch.org/2013/07/24/wipo-talks-on-future-of-tk-genetic-resources-folklore-intensify/> accessed 9 October 2022; Margo Bagley, ‘Of Disclosure Straws and IP System Camels’ in Daniel F Robinson and others (eds), *Protecting Traditional Knowledge: The WIPO Intergovernmental Committee on Intellectual Property and Genetic Resources, Traditional Knowledge and Folklore* (Routledge 2017) 86.

181 WIPO, ‘Report Adopted by the Committee’, WIPO/GRTKF/IC/26/8 (March 24 2014).

182 Bagley (n 180).

183 ibid.

184 Patent Law (n 160).

185 Wenting Cheng, ‘The protection of genetic resources in Chinese patent law' (2021) 43 European Intellectual Property Review 248.

186 Patent Law (n 160).

187 The European Patent Office (EPO) and The Ministry of Commerce (MOFCOM), *Third Revision of China’s Patent Law: Legal Texts and Documents on the Drafting Process 2006–2008* (EU- China IPR2, 2009).

188 WTO, ‘The Protection of Traditional Cultural Expressions: Draft Gap Analysis’, WIPO/GRTKF/IC/13/4(b), March 26 2008; WTO, ‘The Protection of Traditional Cultural Expressions: Updated Draft Gap Analysis’, WIPO/GRTKF/IC/37/6, July 20 2018.

189 ibid.

190 ibid.

191 ibid.

192 ibid.

193 R Lakshmi Poorna and others, ‘Preservation and Protection of Traditional Knowledge – Diverse Documentation Initiatives Across the Globe’ (2014) 107 Current Science 1240; MS Ansari, ‘Evaluation of Role of Traditional Knowledge Digital Library and Traditional Chinese Medicine Database in Preservation of Traditional Medicinal Knowledge’ (2016) 36 Journal of Library & Information Technology 73.

194 Institute of Information on Traditional Chinese Medicine (IITCM), ‘TCM Database System’ (2014) <http://www. cintcm.ac.cn> accessed 14 October 2022.

195 Yanhuai Liu and Yanling Sun, ‘China Traditional Chinese Medicine (TCM) Patent Database’ (2004) 26 World Patent Information 91.

196 Xiulan Zuo, ‘Transnational Curriculum Inquiry’ (2007) 4 Journal of the International Association for the Advancement of Curriculum Studies 80.

197 Juan Wang, ‘National Language Movement - An Indispensable Perspective of Literary Revolution’ (2018) 17 Studies in Literature and Language 114.

198 Chidi Oguamanam, ‘Patents and Traditional Medicine: Digital Capture, Creative Legal Interventions and the Dialectics of Knowledge Transformation’ (2008) 15 Indiana Journal of Global Legal Studies 505.

199 Chinese State Council, ‘Several Opinions on Supporting and Promoting the Development of TMK’ (2009).

200 John Weeks, ‘Chinese TCM Renaissance and the Global Movement for Integrative Health and Medicine’ (2017) 23 The Journal of Alternative and Complementary Medicine 79; Jingfeng Cai, ‘Integration of Traditional Chinese Medicine with Western Medicine—Right or Wrong?’ (1988) 27 Social Science & Medicine 521.

201 John K. Fairbank, Alexander Eckstein and Lien-sheng Yang, ‘Economic Change in Early Modern China: An Analytical Framework’ (1960) 9 Economic Development and Cultural Change 1.

202 Van Dongen, ‘Confucianism, Community, Capitalism’ in Tze-ki Hon and Kristin Stapleton (eds), *Confucianism for the Contemporary World: Global Order, Political Plurality, and Social Action* (SUNY Press 2017) 19.

203 Yu-chih Chen, ‘Chinese Values, Health and Nursing’ (2001) 36 Journal of Advanced Nursing 270; Joanna H Raven and others, ‘Traditional Beliefs and Practices in the Postpartum Period in Fujian Province, China: A Qualitative Study’ (2007) 7 BMC Pregnancy and Childbirth 1.

204 Chenyang Li, ‘Confucian Perspectives on Science and Technology’ (2013) 1 Ethics, Science, Technology, and Engineering: An International Resource, (2013) 1; Anne D Birdwhistell, ‘Medicine and History as Theoretical Tools in a Confucian Pragmatism’ (1995) 45 Philosophy East and West 1.

205 D Gareth Jones and Jing-Bao Nie, ‘Does Confucianism Allow for Body Donation?’ (2018) 11 Anatomical Sciences Education 3; Kwon Ivo, ‘Confucianism Perspectives on Abortion’ in Alireza Bagheri (eds), *Abortion: Global Positions and Practices, Religious and Legal Perspectives* (Springer 2021) 217.

206 A Pang-White, ‘Caring in Confucian Philosophy’ (2011) 6 Philosophy Compass 374.

207 ibid.

208 Anwen Zheng, ‘Ren (仁), The Benevolent Thought of Traditional Chinese Medicine’ (2019) 2 Chinese Medicine and Culture 137.

209 Simiao Sun, ‘Prescriptions for Emergency Worth a Thousand Pieces of Gold (in Chinese)’ in Chinese Medical Association (ed), *A Hundred Must Read Chinese Medicine Classics (in Chinese)* (Hua Xia Press 2008) 9–11.

210 Renzong Qiu, ‘Confucianism, Traditional and Contemporary China’ in Robert M Veatch (ed), *Cross-cultural Perspectives in Medical Ethics* (Jones & Bartlett Publishers 2000) 292.

211 ibid.

212 Weiming Tu, ‘Implications of the Rise of Confucian East Asia’ in Shmuel N Eisenstadt (ed), *Multiple Modernities* (MIT Press 2019) 195; Laurence Jacobs and others, ‘Confucian Roots in China: A Force for Today’s Business’ (1995) 33 Management Decision 29.

213 Huaqiang Zhai and others, ‘The Training Model of Ancient Chinese Medicine Talents Based on the Inheritance of Master and Apprentice and Family Inheritance’ (2014) 20 Chinese Journal of Basic Medicine in Traditional Chinese Medicine 37; Tianbao Qin, ‘Common Pools of Traditional Chinese Medical Knowledge in China’ in Evanson Chege Kamau and Gerd Winter (eds), *Common Pools of Genetic Resources* (Routledge 2013) 150; Dewen Ma ‘Analysis on Cultural Factors Concerning How Patients in China’s Western Ethnic Minority Communities Seek Medical Treatment’ (2017) 54 Journal of Northwest Normal University 71.

214 Wei Shi, ‘Cultural Perplexity in Intellectual Property: Is Stealing a Book an Elegant Offense?’ (2006) 32 North Carolina Journal of International Law 1.

215 Jonathan R Sills, ‘China’s Copyright Crisis: How the Internet has Exacerbated China’s Already Weak Copyright Laws and How the U.S. Can Combat the Pirating of its Software’ (2006) Connecticut Intellectual Property Notes <http://www.law.uconn.edu/journals/cipn/browse.html> accessed 11 October 2022.

216 R Keith Sawyer, ‘The Western Cultural Model of Creativity: Its Influence on Intellectual Property Law’ (2011) 86 Notre Dame Law Review 2029.

217 Marci Hamilton, ‘The TRIPs Agreement: Imperialistic, Outdated, and Overprotective’ (1996) Vanderbilt Journal of Transnational Law 613.

218 J Sundaram, ‘Brazil’s Implementation of TRIPS Flexibilities: Ambitious Missions, Early Implementation, and the Plans for Reform’ (2014) 23 Information & Communications Technology Law 81.

219 Ishan Parashar, ‘Philosophical Foundation of Intellectual Property’ (2020) 21 Supremo Amicus 484; Michael A Kanning, ‘A Philosophical Analysis of Intellectual Property: In defense of Instrumentalism’ (2008) 8 Scholars Commons University of Florida 1; Neil Wilkof, ‘Theories of Intellectual Property: Is it Worth the Effort?’ (2014) 9 Journal of Intellectual Property Law & Practice 257.

220 Jeanne C Fromer, ‘Expressive Incentives in Intellectual Property’ (2012) 98 Virginia Law Review 1745.

221 Estelle Derclaye and Tim Taylor, ‘Happy IP: Replacing the Law and Economics Justification for Intellectual Property Rights with a Well-being Approach’ (2015) 37 European Intellectual Property Review 197; Lisa P Ramsey, ‘Intellectual Property Rights in Advertising’ (2006) 12 Michigan Telecommunications and Technology Law Review 189.

222 Charles R Stone, ‘What Plagiarism Was Not: Some Preliminary Observations on Classical Chinese Attitudes Toward What the West Calls Intellectual Property’ (2008) 92 Marquette Law Review 199; Andrew Evans, ‘Taming the Counterfeit Dragon: The WTO, TRIPS and Chinese Amendments to Intellectual Property Laws’ (2003) 31 Georgia Journal of International & Comparative Law 587.

223 Yu (n 164).

224 Liangnian Jin, *Annotated Translation of the Analects of Confucius* (Shanghai Ancient Books Publishing House 2004) 36.

225 Shaohua Hu, ‘Confucianism and Contemporary Chinese Politics’ (2007) 35 Politics & Policy 136.

226 Joan Trullén and Rafael Boix, ‘Knowledge Externalities and Networks of Cities in the Creative Metropolis’ in Philip Cooke and Luciana Lazzeretti (eds), *Knowledge Externalities and Networks of Cities in the Creative Metropolis* (Edward Elgar Publishing 2007) 211; Luciana Lazzeretti and others, ‘Do Creative Industries Cluster? Mapping Creative Local Production Systems in Italy and Spain’ (2008)15 Industry and Innovation 549.

227 Christopher Ford, *The Mind of Empire: China’s History and Modern Foreign Relations* (University Press of Kentucky 2010) 11.

228 ibid.

229 Alford (n 38).

230 ibid. The original text was ‘Shuer Buzuo, Xiner Haogu’ (述而不作, 信而好古) and ‘Wei Ri Yu Huai Ming De’ (惟曰予怀明德).

231 ‘Writing Paper Became Expensive in Luoyang’ (Eastwest Cultural Centre, 2021) <https://www.ewccenter.com/writing-paper-became-expensive-in-luoyang/>; ‘Paper Becomes Expensive in Luoyang—Good Writings Make People Copy Them’ (Chinlingo, 2016) <https://www.chinlingo.com/articles/800106/>

232 Margo A Bagley, ‘The Fallacy of Defensive Protection for Traditional Knowledge’ (2019) 58 Washburn Law Journal 323; Antony Taubman and Matthias Leister, ‘Analysis of Different Areas of Indigenous Resources: Traditional Knowledge’ in Silke von Lewinski (eds), *Indigenous Heritage and Intellectual Property: Genetic Resources, Traditional Knowledge and Folklore* (Kluwer Lae International 2008) 59.

233 Xiaotong Fei, ‘The Formation and Development of the Chinese Nation with Multi-ethnic Groups’ (2017) 1 International Journal of Anthropology and Ethnology 1.

234 Jianzhuo Teng, ‘Discussion on the Holistic Characters of Tujia Medicine’ (2007) 2 Journal of Medicine & Pharmacy of Chinese Minorities 6; Wuritunashu, ‘The Holistic View of Mongolian Medicine and Its Application’ (2014) 5 Journal of Medicine & Pharmacy of Chinese Minorities 58; Ya Zhao, ‘The Discovery of New Drugs for the Treatment of Diabetic Based on the Understanding of the Holistic View of Tibetan Medicine’ (2019) 25 Chinese Journal of Experimental Traditional Medical Formulae 167.

235 Zhaoyun Zhu and others, ‘Innovative Development Path of Ethnomedicines: An Overview of Ethnomedicines in China’ (2016) 10 Frontiers in Medicine 166.

236 ibid.

237 ibid.

238 ibid; Fuming Lee, ‘The Legal Protection of Tibetan Intangible Cultural Heritage in China: From the Perspective of Tibetan Customary Law and Intellectual Property Law’ (2019) 7 China Legal Science 91; Mohammd Ushur, ‘On Restructuring the Chinese IPR Regime from Ethnic Group Prospect’ (2015) 10 Frontiers of Law in China 732.

239 ibid.

240 Geoffrey B. Samuel, ‘Tibetan Medicine and Biomedicine: Epistemological Conflicts, Practical Solutions’ (2006) 2 Asian Medicine 72.

241 ibid.

242 Jane E Anderson, ‘Analysis of Different Areas of Indigenous Resources: Traditional Knowledge’ in Jane E Anderson (ed), *Law, Knowledge, Culture: The Production of Indigenous Knowledge in Intellectual Property Law* (Edward Elgar Publishing 2009) 172.

243 ibid.

244 Jeanne C Fromer, ‘Patent Disclosure’ (2009) 94 Iowa Law Review 539.

245 Johanna Gibson, ‘Intellectual Property and Other Objects of Protection’ in Johanna Gibson (ed), *Community Resources: Intellectual Property, International Trade and Protection of Traditional Knowledge* (Routledge 2005) 101.

246 Maram Suresh Gupta, ‘Sufficiency of Disclosure in Patent Specification’ (2009) 14 Journal of Intellectual Property Rights 307; Jason Rantanen, ‘The Doctrinal Structure of Patent Law’s Enablement Requirement’ (2016) 69 Vanderbilt Law Review 1679.

247 Teresa Scassa and Fraser Taylor, ‘Legal and Ethical Issues Around Traditional Knowledge in Polar Data Infrastructures’ (2017) 16 Data Science Journal 1.

248 Catherine Bell and Caeleigh Shier, ‘Control of Information Originating from Aboriginal Communities: Legal and Ethical Contexts’ (2011) 35 Études Inuit Studies 36.

249 Geoffroy Fauchet, ‘Dai Medicine: Preservation of and Changes in Ancient Healing Practices’ (2006) 621 Independent Study Project (ISP) Collection 1; Zhiyong Li and others, ‘Policies and Problems of Modernizing Ethnomedicine in China: A Focus on the Yi and Dai Traditional Medicines of Yunnan Province’ (2020) Evidence-Based Complementary and Alternative Medicine 1; Eric J Buenz, Rob Verpoorte and Brent A Bauer, ‘The Ethno-pharmacologic Contribution to Bioprospecting Natural Products’ (2018) 58 Annual Review of Pharmacology and Toxicology 509.

250 Harilal Madhavan, ‘Below the Radar Innovations and Emerging Property Right Approaches in Tibetan Medicine’ (2017) 20 The Journal of World Intellectual Property 239.

251 Patent Law (n 160) ‘inventiveness means that, as compared with the technology existing before the date of filing, the invention has prominent substantive features and represents a notable progress’; Xuan Li and Weiwei Li, ‘Inadequacy of Patent Regime on Traditional Medicinal Knowledge—A Diagnosis of 13‐Year Traditional Medicinal Knowledge Patent Experience in China’ (2007) 10 The Journal of World Intellectual Property 125.

252 Deepa Varadarajan, ‘A Trade Secret Approach to Protecting Traditional Knowledge’ (2011) 36 The Yale Journal of International Law 371.

253 Gibson (n 245).

254 Patent Law (n 160).

255 Mohammed Rafiquzzaman, ‘The Optimal Patent Term Under Uncertainty’ (1987) 5 International Journal of Industrial Organization 233.

256 ibid.

257 Zhihong Wang and Xinwei Xiang, ‘Thoughts on the Protection, Inheritance and Development of Traditional Medicine Culture of Ethnic Minorities’ (2012) 35 Journal of Yunnan University of Traditional Chinese Medicine 1.

258 Manuel Ruiz, ‘The International Debate on Traditional Knowledge as Prior Art in the Patent System: Issues and Options for Developing Countries’ (2006) Centre for International Environmental Law 1.

259 Tianbao Qin, ‘Common Pools of Traditional Chinese Medical Knowledge in China’ in Evanson Chege Kamau and Gerd Winter (eds), *Common Pools of Genetic Resources* (Routledge 2013) 150.

260 Tsutomu Inamura, ‘Traditional Knowledge and Chinese Traditional Medicine Concerning ABS: Political Mistranslation by Chinese Government and Japanese Government’ (17th Conference of the Science Council of Asia, 2017) 1.

261 Tony Chui, ‘Secret Medicine in the Writings of Sanggyé Gyatso: Encoded Esoteric Material of Therapeutics’ in William A McGrath (eds), *Knowledge and Context in Tibetan Medicine* (Brill Academic Publishers 2019) 85.

262 ibid.

263 Patent Law (n 160); Chinese Patent Examination Guidelines, No 391, Promulgated by State Intellectual Property Office on 11 December 2020 (The Guidelines) 153.

264 Qin (n 259).

265 Sijia Liu ‘New Law Sparks the Expectation Over the Future of Traditional Chinese Medicine: Can TCM Law Effectively Promote the Development of TCM Industry in China?’ (2018) 37 Medicine and Law 193.

266 Siyuan Pan and others, ‘Traditional Medicines in the World: Where to Go Next?’ (2014) 2014 Evidence-Based Complementary and Alternative Medicine 1.

267 The Guidelines (n 263).

268 Li and Li (n 251).

269 ibid.

270 The Guidelines (n 263).

271 Li and Li (n 268).

272 Patent Law (n 160).

273 Qin (n 259).

274 ibid.

275 ibid.

276 ibid.

277 Li and Li (n 268).

278 Samuel L Myers and others, ‘Ethnic Minorities, Race, and Inequality in China: A New Perspective on Racial Dynamics’ (2013) 40 Review of Black Political Economy 231.

279 Colin Mackerras ‘The Economies of the Minorities’ in Colin Mackerras (ed), *China’s Ethnic Minorities and Globalisation* (Routledge 2003) 56.

280 Sumathi Subbiah, ‘Reaping What They Sow: The Basmati Rice Controversy and Strategies for Protecting Traditional Knowledge’ (2004) 27 Boston College International and Comparative Law Review 529.

281 Lanrong Xue and Tianjian Shi, ‘Inequality in Chinese Education’ (2001) 10 Journal of Contemporary China 107.

